# Families of stable solitons and excitations in the PT-symmetric nonlinear Schrödinger equations with position-dependent effective masses

**DOI:** 10.1038/s41598-017-01401-3

**Published:** 2017-04-28

**Authors:** Yong Chen, Zhenya Yan, Dumitru Mihalache, Boris A. Malomed

**Affiliations:** 1 Key Laboratory of Mathematics Mechanization, Institute of Systems Science, AMSS, Chinese Academy of Sciences, Beijing, 100190 China; 2 School of Mathematical Sciences, University of Chinese Academy of Sciences, Beijing, 100049 China; 3 Department of Theoretical Physics, Horia Hulubei National Institute of Physics and Nuclear Engineering, PO Box MG-6, Bucharest, Romania; 4 Department of Physical Electronics, School of Electrical Engineering, Faculty of Engineering, Tel Aviv University, Tel Aviv, 59978 Israel; 5 Laboratory of Nonlinear-Optical Informatics, ITMO University, St. Petersburg, 197101 Russia

## Abstract

Since the parity-time-($\pmb{\mathscr{P}}\pmb{\mathscr{T}}$-) symmetric quantum mechanics was put forward, fundamental properties of some linear and nonlinear models with $\pmb{\mathscr{P}}\pmb{\mathscr{T}}$-symmetric potentials have been investigated. However, previous studies of $\pmb{\mathscr{P}}\pmb{\mathscr{T}}$-symmetric waves were limited to constant diffraction coefficients in the ambient medium. Here we address effects of variable diffraction coefficient on the beam dynamics in nonlinear media with generalized $\pmb{\mathscr{P}}\pmb{\mathscr{T}}$-symmetric Scarf-II potentials. The broken linear $\pmb{\mathscr{P}}\pmb{\mathscr{T}}$ symmetry phase may enjoy a restoration with the growing diffraction parameter. Continuous families of one- and two-dimensional solitons are found to be stable. Particularly, some stable solitons are analytically found. The existence range and propagation dynamics of the solitons are identified. Transformation of the solitons by means of adiabatically varying parameters, and collisions between solitons are studied too. We also explore the evolution of constant-intensity waves in a model combining the variable diffraction coefficient and complex potentials with globally balanced gain and loss, which are more general than $\pmb{\mathscr{P}}\pmb{\mathscr{T}}$-symmetric ones, but feature similar properties. Our results may suggest new experiments for $\pmb{\mathscr{P}}\pmb{\mathscr{T}}$-symmetric nonlinear waves in nonlinear nonuniform optical media.

## Introduction

The Hamiltonians in the quantum mechanics are usually required to be Hermitian, which secures the corresponding spectra to be real^1^. Nevertheless, it had been demonstrated by Bender and Boettcher in 1998 that non-Hermitian Hamiltonians obeying the parity-time (${\mathscr{P}}{\mathscr{T}}$) symmetry may also produce entirely real spectra^2–8^. The ${\mathscr{P}}{\mathscr{T}}$ symmetry implies that the real and imaginary parts of the complex-valued potential, $U(x)=V(x)+iW(x)$, are, respectively, even and odd functions of the coordinate^2^: *V*(*x*) = *V*(−*x*), *W*(−*x*) = −*W*(*x*). For a given real part of the potential, the spectrum of most ${\mathscr{P}}{\mathscr{T}}$-symmetric systems remains real, as long as the amplitude of the imaginary component of the potential is kept below a certain critical value (the ${\mathscr{P}}{\mathscr{T}}$-symmetry-breaking threshold); nevertheless, dynamical models featuring unbreakable ${\mathscr{P}}{\mathscr{T}}$ symmetry are known too^9^. Pioneering theoretical works had predicted a possibility to realize the ${\mathscr{P}}{\mathscr{T}}$-symmetric wave propagation in optical media with symmetrically placed gain and loss elements^10–14^, which was followed by the experimental implementation in optical and atomic settings, including synthetic photonic lattices, metamaterials, microring lasers, whispering-gallery microcavities, and optically induced atomic lattices^15–22^. In particular, phase transitions between regions of the unbroken and broken ${\mathscr{P}}{\mathscr{T}}$ symmetry have been observed in many experiments.

On the theoretical side, the consideration of ${\mathscr{P}}{\mathscr{T}}$-symmetric potentials in both one- and multi-dimensional linear and nonlinear Schrödinger (NLS) or Gross-Pitaevskii (GP) equations has revealed many remarkable ${\mathscr{P}}{\mathscr{T}}$-symmetry-breaking phenomena, including several models that give rise to ${\mathscr{P}}{\mathscr{T}}$-symmetric solitons^23–52^. It is commonly known that the soliton theory has been widely applied to fluid mechanics, plasma physics, Bose-Einstein condensates (BECs), nonlinear optics, and many other fields. In particular, optical solitons can utilize the nonlinearity in optical fibers to balance the group-velocity dispersion, thus stably propagating in long-scale telecommunication links. More recently, stable ${\mathscr{P}}{\mathscr{T}}$-symmetric solitons were also investigated in the third-order NLS equation^53^, the generalized GP equation with a variable group-velocity coefficient^54^, and the derivative NLS equation^55^. The vast work performed in the field of nonlinear waves in ${\mathscr{P}}{\mathscr{T}}$-symmetric systems has been summarized in two recent comprehensive reviews^56, 57^.

As mentioned above^2^, the usual one-dimensional (1D) ${\mathscr{P}}{\mathscr{T}}$-symmetric Hamiltonian is ${\mathscr H} =-{\partial }_{x}^{2}+V(x)+iW(x)$, with *V*(−*x*) = *V*(*x*) and *W*(−*x*) = −*W*(*x*). However, physics of semiconductors gives rise to a Hamiltonian in which the effective mass of collective excitations^58^, *M*(*x*), may be variable (position-dependent)^59–66^: ${ {\mathscr H} }_{M}=-\frac{1}{2}{\hslash }^{2}{\partial }_{x}(\frac{1}{M(x)}{\partial }_{x})+V(x)$.

To the best of our knowledge, Hamiltonians combining a variable effective mass and complex-valued ${\mathscr{P}}{\mathscr{T}}$-symmetric potentials, in particular, ${ {\mathscr H} }_{M}^{({\mathscr{P}}{\mathscr{T}})}=-\frac{1}{2}{\hslash }^{2}{\partial }_{x}(\frac{1}{M(x)}{\partial }_{x})+V(x)+iW(x)$, have not been studied yet. In fact, this model represents more general settings than the above-mentioned one occurring in semiconductors. Indeed, the effective mass for collective excitations propagating in lattice media is determined by local properties of the underlying lattice^67, 68^, which may be nonuniform in many situations, thus making the effective mass position-dependent. In the context of optics, essentially the same Hamiltonian governs the light propagation in the spatial domain, where the effective diffraction coefficient, 1/*M*, can be also made *x*-dependent in nonuniform photonic lattices^69, 70^.

The goal of the present work is to introduce 1D and 2D NLS models with such Hamiltonians, incorporating a particular physically relevant ${\mathscr{P}}{\mathscr{T}}$-symmetric potential, namely, a generalized Scarf-II potentials (i.e., a hyperbolic version of the quantum-mechanical potential introduced by Scarf ^71^). We reveal characteristic properties of both 1D and 2D linear and nonlinear modes in such models, including solitons in the case of cubic nonlinearity, which are quite generic and can be extended to other physically relevant complex-valued ${\mathscr{P}}{\mathscr{T}}$-symmetric potentials.

## Results

### 1D $\pmb{\mathscr{P}}\pmb{\mathscr{T}}$-symmetric nonlinear waves in the effective diffraction

In Kerr-type nonlinear media with the complex-valued ${\mathscr{P}}{\mathscr{T}}$-symmetric potential and the effective diffraction coefficient defined by the position-dependent mass, $m(x)\equiv 1/M(x)$ (in particular, it represents the above-mentioned variable diffraction coefficient in optics), the scaled 1D modified NLS equation for the wave function $\psi (x,z)$ is

1 $$i\frac{\partial \psi }{\partial z}=[-\frac{\partial }{\partial x}(m(x)\frac{\partial }{\partial x})+V(x)+iW(x)-g{|\psi |}^{2}]\psi ,$$

where *z* is the propagation distance, *x* is the transverse coordinate, and *g* is the strength of the Kerr nonlinearity. Replacing *z* by time *t*, Eq. (1) may be used as the GP equation for the BEC wave function, with the effective mass affected by a nonuniform optical lattice^72^. In the spatial-domain optics, real potential *V*(*x*) represents the local modification of the refractive index, whereas *W*(*x*) stands for the transverse symmetric distribution of the optical gain (*W* > 0) and loss (*W* < 0). Under the conditions that *V*(*x*) + *iW*(*x*) is a ${\mathscr{P}}{\mathscr{T}}$-symmetric potential and *m*(*x*) is an even function of *x*, it is easy to see that Eq. (1) is invariant under the action of ${\mathscr{P}}{\mathscr{T}}$-symmetric transformation, where the spatial-reflection operator ${\mathscr{P}}$ and time-reversal operator ${\mathscr{T}}$ are defined as usual^2^, ${\mathscr{P}}:x\to -x;{\mathscr{T}}:i\to -i,z\to -z$. Equation (1) may be rewritten in the variational form, $i{\psi }_{z}=\delta {\mathscr{H}}(\psi )/(\delta {\psi }^{\ast })$, with a non-Hermitian but ${\mathscr{P}}{\mathscr{T}}$-symmetric field Hamiltonian, ${\mathscr H} (\psi )={\int }_{-\infty }^{+\infty }\{m(x){|{\psi }_{x}|}^{2}+[V(x)+iW(x)]{|\psi |}^{2}-\frac{g}{2}{|\psi |}^{4}\}dx$, where the asterisk denotes the complex conjugate. Further, the power (norm) of the wave function, $P(z)={\int }_{-{\rm{\infty }}}^{+{\rm{\infty }}}{|\psi (x,z)|}^{2}dx$, evolves according to equation $dP/dz=2{\int }_{-{\rm{\infty }}}^{+{\rm{\infty }}}W(x){|\psi |}^{2}dx$, which can be immediately deduced from Eq. (1).

### Linear spectra of unbroken and broken $\pmb{\mathscr{P}}\pmb{\mathscr{T}}$-symmetric phases

We now introduce the diffraction coefficient with a localized spatial modulation,

2 $$m(x)={m}_{\alpha }(x)={m}_{0}\,{\rm sech }^{\alpha }\,x+1,$$

and a physically relevant ${\mathscr{P}}{\mathscr{T}}$-symmetric ingredient of the model in the form of a generalized Scarf-II potential

3 $${V}_{\alpha }(x)={{\mathscr{V}}}_{1}\,{\rm sech }^{2}\,x+{{\mathscr{V}}}_{2}\,{\rm sech }^{\alpha }\,x,$$

4 $${W}_{\alpha }(x)=(\tanh \,x)({\rm sech }\,x)\,[{{\mathscr{W}}}_{1}+{{\mathscr{W}}}_{2}\,{\rm sech }^{\alpha }\,x],$$

where *m*_0_ > −1 and *α* > 0 are both real constants, ${{\mathscr{V}}}_{1}=-\frac{1}{4}\mathrm{(4}{\theta }_{0}^{2}+{\alpha }^{2}+6\alpha +\mathrm{8)}$, ${{\mathscr{V}}}_{2}=\frac{{m}_{0}}{4}\mathrm{(3}{\alpha }^{2}+8\alpha +\mathrm{4)}$, ${{\mathscr{W}}}_{1}=-{\theta }_{0}(\alpha +\mathrm{3)}$, and ${{\mathscr{W}}}_{2}=-{m}_{0}{\theta }_{0}\mathrm{(2}\alpha +\mathrm{3)}$. Here *m*_0_ and *θ*_0_ account for the modulation of the local diffraction coefficient *m*_*α*_, even real potential *V*_*α*_, and odd gain-loss distribution *W*_*α*_, respectively. For *m*_0_ = 0, Eq. (2) yields a constant diffraction coefficient, *m* = 1, hence Eq. (1) reduces to the usual NLS equation. The corresponding complex-valued potential (3) and (4) becomes the usual Scarf-II potential: $U(x)={{\mathscr{V}}}_{1}\,{\rm sech }^{2}\,x+i{{\mathscr{W}}}_{1}\,{\rm sech}\,x\,\tanh \,x$ ^73^, where the corresponding Hamiltonian $H(x)=-{\partial }_{x}^{2}+U(x)$ may have two branches of energy eigenvalues and interpreted as the so-called quasi-parity^74^. Moreover, the Hamiltonian *H*(*x*) can be shown to exhibit the spontaneous ${\mathscr{P}}{\mathscr{T}}$-symmetry breaking when the strength of the imaginary part, $|{{\mathscr{W}}}_{1}|$, exceeds a threshold $-{{\mathscr{V}}}_{1}+\mathrm{1/4}$ ^75^. Below, we use the complex potential with *m*_0_ > 0 and *α* > 0, which may be viewed as a generalization of the basic Scarf-II potential.

First, we consider linear spectra of phases with unbroken and broken ${\mathscr{P}}{\mathscr{T}}$ symmetries in the framework of the linear spectral problem with ${\mathscr{P}}{\mathscr{T}}$-symmetric operator $\hat{L}$ that includes variable diffraction coefficient (2) and ${\mathscr{P}}{\mathscr{T}}$-symmetric potential (3) and (4). The problem is based on the equation for localized eigenfunctions ${\rm{\Phi }}(x)$ and respective eigenvalues *λ*,

5 $$\hat{L}{\rm{\Phi }}(x)=\lambda {\rm{\Phi }}(x),\,\hat{L}\equiv -\frac{d}{dx}(m(x)\frac{d}{dx})+V(x)+iW(x\mathrm{).}$$

**Figure 1:**
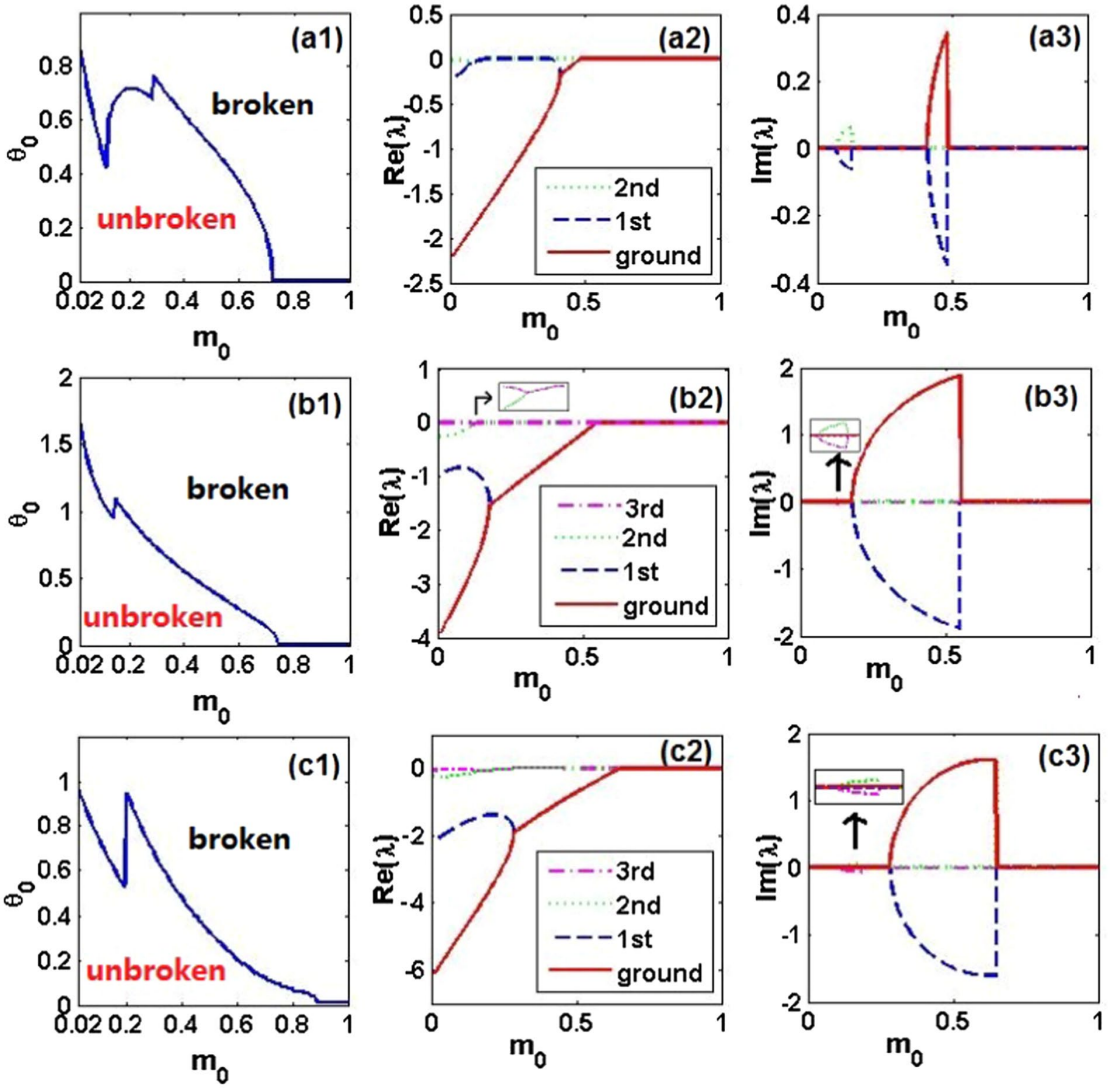
Linear spectrum in ${\mathscr{P}}{\mathscr{T}}$-symmetric potentials. (**a1**,**b1**,**c1**) The ${\mathscr{P}}{\mathscr{T}}$-symmetry of states produced by linear eigenvalue problem (5), with the position-dependent diffraction coefficient (2) and generalized ${\mathscr{P}}{\mathscr{T}}$-symmetric Scarf-II potentials (3) and (4), is unbroken/broken in the domains below/above the solid blue curves. The results are displayed for *α* = 1, 2, 3, respectively. (**a2**,**b2**,**c2**) Real and (**a3**,**b3**,**c3**) imaginary parts of first several eigenvalues *λ* as a function of *m*_0_ for *θ*_0_ = 0.6, 1, 0.7, respectively. The insets in (**b2**) and (**b3**,**c3**) show, severally, the separation and coalescence of the real and imaginary parts of the second and third energy eigenvalues.

In the limit of *m*_0_ = 0 in Eq. (2), $\hat{L}$ amounts to the standard Hamiltonian operator with the usual ${\mathscr{P}}{\mathscr{T}}$-symmetric Scarf-II potential, which has been studied in detail by means of analytical and numerical methods^54, 75^. For *m*_0_ > 0, we focus on natural values of powers in Eq. (2), *α* = 1, 2, and 3. By means of the available numerical Fourier spectral algorithm^76, 77^, we find symmetry-breaking boundaries in the (*m*_0_, *θ*_0_) parameter plane, below and above which the ${\mathscr{P}}{\mathscr{T}}$ symmetry is unbroken and broken, respectively, as shown in Figs. 1(a1,b1,c1)). It is seen that, for fixed *m*_0_, there always exists a critical values of *θ*_0_, beyond which the symmetry-breaking phase transition makes the spectra complex-valued. On the other hand, for given *θ*_0_, the phase transition may occur more than once with the increase of *m*_0_, featuring transitions of the energy spectra first from real to complex, then back to real (*restoration* of the ${\mathscr{P}}{\mathscr{T}}$ symmetry), and finally again from real to complex values. This scenario is drastically different from what is known in the case of the usual Hamiltonian with the ${\mathscr{P}}{\mathscr{T}}$-symmetric Scarf-II potential^75^. To illustrate these findings, a few lowest energy levels are displayed in Figs. 1(a2,b2,c2) (real parts) and Figs. 1(a3,b3,c3) (imaginary parts).

### Nonlinear localized modes and their instability

For the given spatial profile of the diffraction coefficient (2) and the generalized ${\mathscr{P}}{\mathscr{T}}$-symmetric Scarf-II potential (3) and (4), it is possible to find analytically particular *exact solutions* for stationary nonlinear localized modes (bright solitons) of Eq. (1) for *g* > 0 in Eq. (1), i.e., the self-focusing sign of the cubic nonlinearity (see Methods):

6 $$\psi (x,z)=\rho \,{\rm sech }^{\frac{\alpha +2}{2}}x\,\exp [i{\theta }_{0}\,{\tan }^{-1}(\sinh \,x)+i\mu z],$$

where $\rho =\sqrt{{m}_{0}\mathrm{(4}{\theta }_{0}^{2}+3{\alpha }^{2}+10\alpha +\mathrm{8)/(4}g)}$, and the propagation constant is $-\mu =-{(\frac{\alpha }{2}+1)}^{2}$. Without loss of generality, we display subsequent results for the normalization defined by *g* = 1.

The integral power of the nonlinear localized modes (6) is $P={\int }_{-\infty }^{+\infty }{|\psi (x,z)|}^{2}dx={\rho }^{2}{\int }_{-\infty }^{+\infty }{\rm sech }^{\alpha +2}xdx$, which is $\frac{1}{2}\pi {\rho }^{2}$, $\frac{4}{3}{\rho }^{2}$, $\frac{3}{8}\pi {\rho }^{2}$ for *α* = 1, 2, 3, respectively. It is also relevant to examine the transverse power flow of these modes (alias the Poynting vector), $S(x)=(i\mathrm{/2})(\varphi {\varphi }_{x}^{\ast }-{\varphi }^{\ast }{\varphi }_{x})={\rho }^{2}{\theta }_{0}\,{\rm sech }^{\alpha +3}(x)$, whose sign is solely determined by *θ*_0_ for any *α* > 0. It is clearly seen from Eq. (4) that the signs of gain-loss distribution *W*_*α*_ are also determined by the single parameter *θ*_0_, for *m*_0_ ≥ 0 and *α* > 0. Thus we conclude that the power always flows from the gain region to one carrying the loss, regardless of the sign of *θ*_0_.

**Figure 2:**
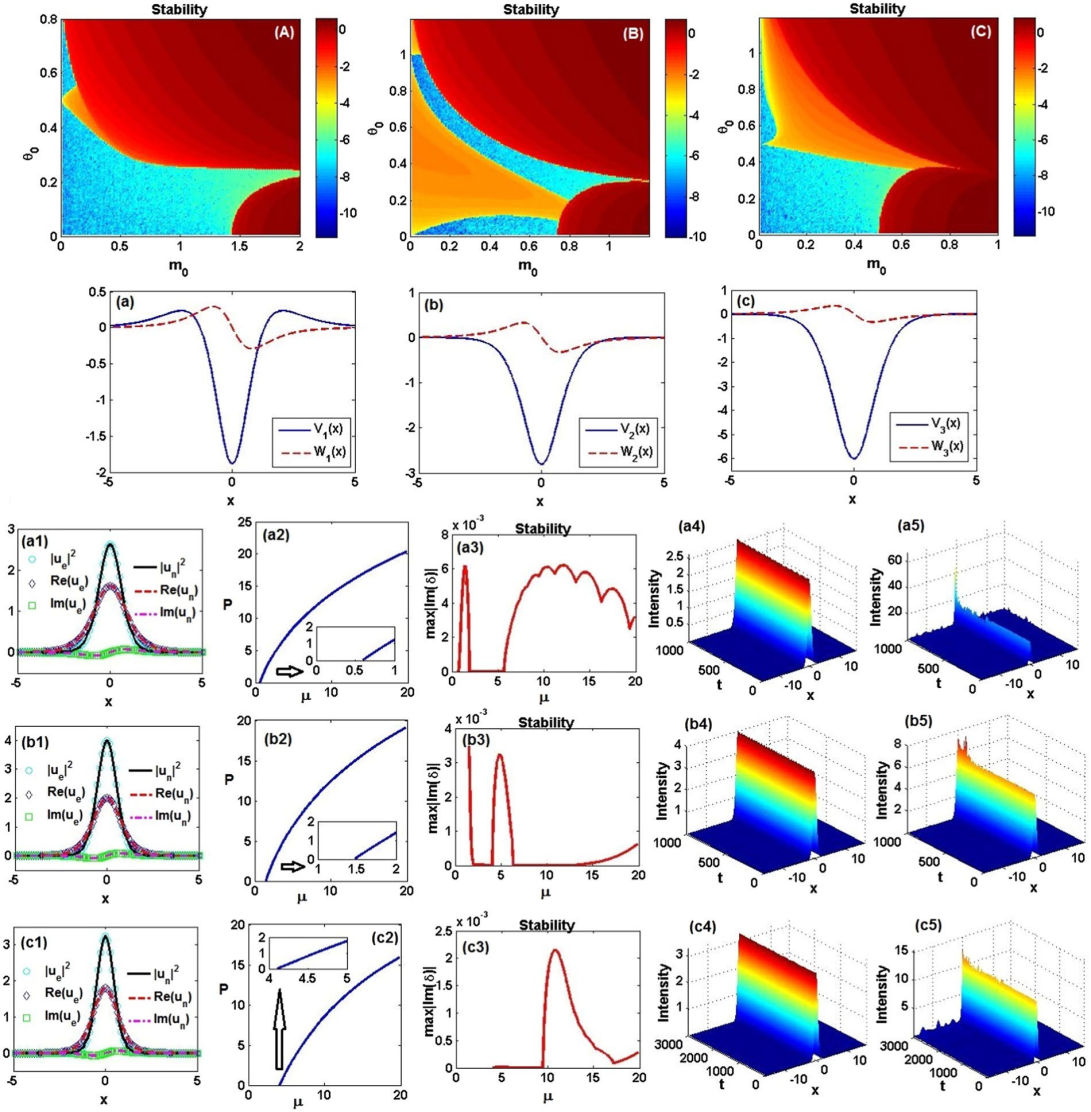
Stability of exact nonlinear modes (6). (**A**–**C**) The linear-stability map, as obtained from the numerical solution of Eq. (23), in the $({m}_{0},{\theta }_{0})$ plane for $\alpha =1,2,3$, respectively. The stability is determined by the largest absolute value of imaginary parts of the linearization eigenvalue *δ*, which are displayed by means of the color bar on a common logarithmic scale, log[|(Im(*δ*)|)]. (**a**–**c**) The real and imaginary parts of the ${\mathscr{P}}{\mathscr{T}}$-symmetric complex potentials determined by Eqs. (3) and (4) for (*α*, *m*_0_, *θ*_0_) = (1, 0.5, 0.1), (2, 0.4, 0.1), (3, 0.2, 0.1), respectively. For *α* = 1, 2, 3, which correspond to (**a1**,**b1**,**c1**), respectively, the real parts, imaginary parts, and intensity profiles are displayed, as obtained from the exact solutions, ${\varphi }_{e}$, and their numerically found counterparts (fundamental solitons), ${\varphi }_{n}$, for *μ* = 9/4, 4, 25/4, respectively. (**a2**,**b2**,**c2**) Power *P* versus the frequency, −*μ*. (**a3**,**b3**,**c3**) Linear-stability spectra of numerically found fundamental solitons versus *μ*. (**a4**,**b4**,**c4**) The stable evolution of the solitons (no matter exact or numerical ones, the evolution being identical) from (**a1**,**b1**,**c1**). (**a5**,**b5**,**c5**) Unstable evolution of the numerically found solitons at $\mu =10,\mu =5,\mu =10.5$, respectively. The insets in (**a2**,**b2**,**c2**) indicate power curves near the lower cutoff points of *μ*.﻿ Not﻿e t﻿hat t-axes should be z-axes in (**a4**,**a5**,**b4**,**b5**,**c4**,**c5**).

For different powers *α* = 1, 2, 3, we aim to study the linear stability of exact nonlinear modes (6) by numerically calculating the largest absolute value of imaginary parts of the linearization eigenvalue *δ* from Eq. (23), see below, in the modulation-parameter plane (*m*_0_, *θ*_0_). Figures 2(A–C) exhibit the so found stability (blue) and instability (other colors) regions of the localized modes (6) for *α* = 1, 2, 3, respectively. For these three cases, we, respectively, choose three stable points (i.e., ones with the unbroken ${\mathscr{P}}{\mathscr{T}}$ symmetry): $({m}_{0},{\theta }_{0})=\mathrm{(0.5},\mathrm{0.1)},\mathrm{(0.4},\mathrm{0.1)},\mathrm{(0.2},\mathrm{0.1)}$, to display the corresponding ${\mathscr{P}}{\mathscr{T}}$-symmetric potentials in Figs. 2(a–c). The single-well potential becomes deeper, whereas the amplitude of the gain-and-loss distribution slowly decreases, as *α* increases and *m*_0_ decreases. For these three cases, we show exact solitonic solutions (6) and their numerically found counterparts in Figs. 2(a1,b1,c1), to corroborate that they are mutually identical. For other values of propagation constant −*μ*, exact analytical solutions are not available, but we can use the numerical method (validated by the comparison with the exact solutions) to produce fundamental solitons. It follows from Figs. 2(a2,b2,c2) that the solitons’ powers are monotonously increasing functions of *μ*. While existence ranges of the numerically found solitons have nearly the same upper cutoff ${\mu }_{{\rm{up}}}\approx 19.8$, the lower cutoffs are different: ${\mu }_{{\rm{1low}}}=0.6$, ${\mu }_{{\rm{2low}}}=1.48$, ${\mu }_{{\rm{3low}}}=4.11$, respectively, with a trend to growth. In the existence range of the numerically found solitons, the dependence of the corresponding linear-stability eigenvalues (the largest absolute value of the imaginary part of the perturbation growth rate *δ*) on the propagation constant −*μ* is shown in Figs. 2(a3,b3,c3). It is seen that the numerically found soliton solutions in Figs. 2(a1,b1,c1), corresponding to *μ* = 2.25, 4, 6.25, respectively, are completely stable, in accordance with the stability of the corresponding exact nonlinear modes (see Figs. 2(A–C)).

To validate the linear-stability results, we have simulated the propagation, by taking the input provided by the stationary modes in Figs. 2(a1,b1,c1) with the addition of 2% random perturbations, as is shown in Figs. 2(a4,b4,c4), respectively. In practice, we simulate the beam propagation with the initial input $\psi (x,z=0)=\varphi (x\mathrm{)(1}+\epsilon )$, where $\varphi (x)$ is a nonlinear mode, and $\epsilon$ is a complex broadband random perturbation. In MATLAB, the 2% white noise $\epsilon$ can be realized by utilizing a random matrix such as $\epsilon =\mathrm{0.04(}{\rm{rand}}(N,1)-0.5)\,(1+i)/\sqrt{2}$, where rand(*N*, 1) is an *N*–*by*–1 array of pseudorandom uniform values on the open interval (0, 1) (similarly in other cases, even for the 2D situation). Finally, based on the linear stability results presented in Figs. 2(a3,b3,c3), we choose solutions that are predicted to be unstable (with *μ* = 10, 5, 10.5), to test the dynamical behavior of the corresponding numerically found soliton solutions. It is found that they are indeed (weakly) unstable, see Figs. 2(a5,b5,c5).

**Figure 3:**
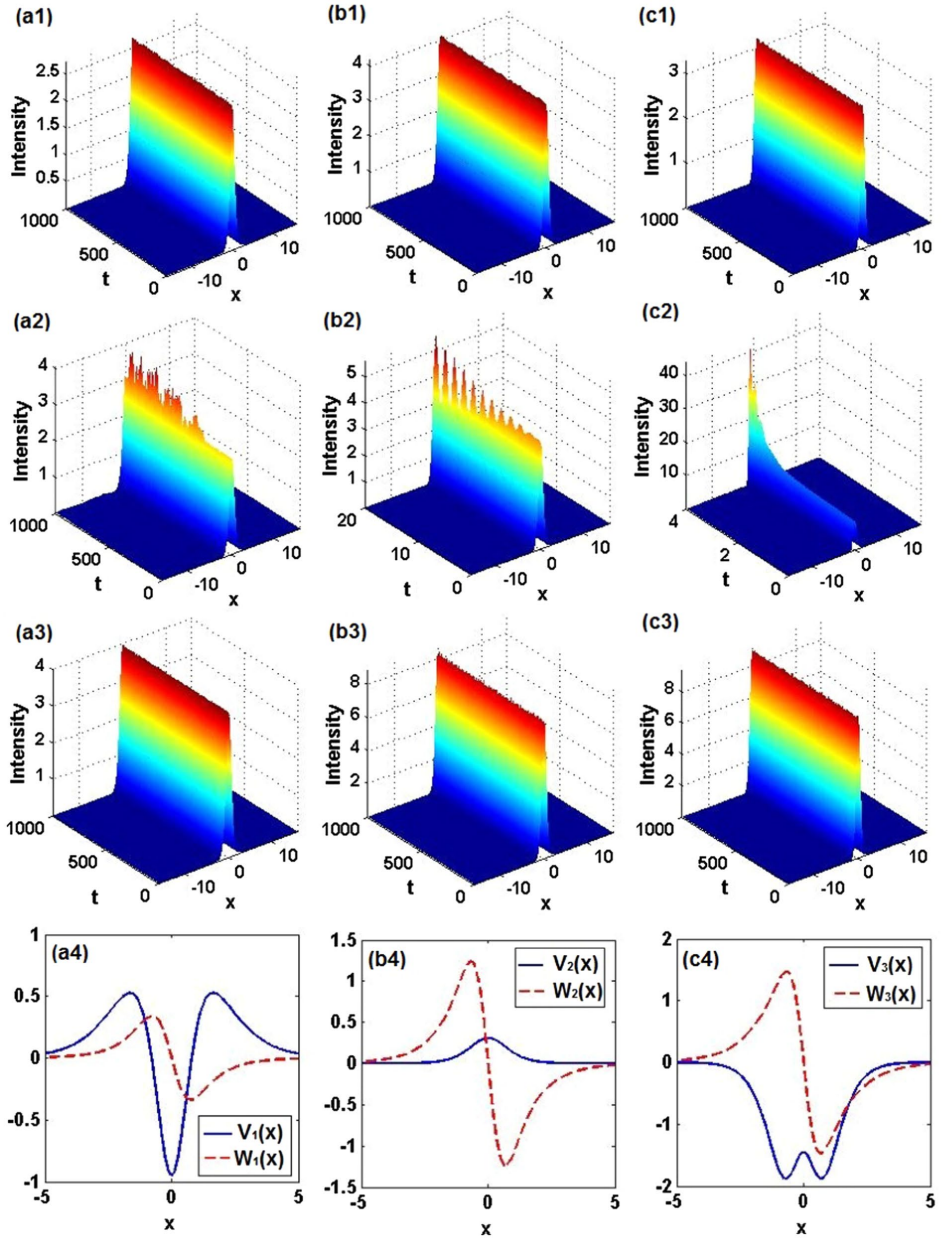
Propagation dynamics of exact nonlinear localized modes (6). (**a1**) ${m}_{0}=0.5,{\theta }_{0}=0.3$, unbroken; (**a2**) ${m}_{0}=0.5,{\theta }_{0}=0.32$, unbroken; (**a3**) ${m}_{0}=0.75,{\theta }_{0}=0.1$, broken; (**b1**) ${m}_{0}=0.4,{\theta }_{0}=0.5$, unbroken; (**b2**) ${m}_{0}=0.4,{\theta }_{0}=0.55$, broken; (**b3**) ${m}_{0}=0.8,{\theta }_{0}=0.3$, broken; (**c1**) ${m}_{0}=0.2,{\theta }_{0}=0.36$, unbroken; (**c2**) ${m}_{0}=0.54,{\theta }_{0}=0.1$, unbroken; (**c3**) ${m}_{0}=0.54,{\theta }_{0}=0.36$, broken. (**a4**,**b4**,**c4**) Real and imaginary parts of the ${\mathscr{P}}{\mathscr{T}}$-symmetric potential corresponding to cases (**a3**,**b3**,**c3**), respectively. Other parameters are, severally, *α* = 1, 2, and 3, for the first, second, and third columns. Note﻿ t﻿hat t-axes﻿ ﻿should be z-axes in (**a1**,**b1**,**c1**,**a2**,**b2**,**c2**,**a3**,**b3**,**c3**).

Further, following the results of the linear-stability analysis shown in Figs. 2(A–C), we have tested the propagation dynamics of the exact nonlinear modes (6), varying the parameter *m*_0_ or *θ*_0_ in Eqs. (2)–(4). For the brevity’s sake, hereafter we use the words “unbroken” and “broken” to mention if the corresponding linear modes, considered above, do or do not keep their ${\mathscr{P}}{\mathscr{T}}$ symmetry. For fixed *α* = 1 and *m*_0_ = 0.5, as *θ*_0_ increases continuously from 0.1 to 0.3 (unbroken), nonlinear modes (6) always feature stable propagation dynamics, as shown in Fig. 3(a1). However, as *θ*_0_ increases to slightly larger values, e.g., 0.32 (unbroken), instability sets in, see Fig. 3(a2). Moreover, we have found that a *stable* nonlinear localized mode (6) with (*m*_0_, *θ*_0_) = (0.75, 0.1) belongs to the region of *broken* linear ${\mathscr{P}}{\mathscr{T}}$-symmetry (see Fig. 3(a3)), with the real part of the corresponding complex potential having a single-well shape, see Fig. 3(a4). The latter result implies that the exact ${\mathscr{P}}{\mathscr{T}}$-symmetric nonlinear modes may be stable while their linear counterparts are not.

For fixed *α* = 2 and *m*_0_ = 0.4, as *θ*_0_ grows continuously from 0.1 to 0.5 (unbroken) and further to 0.55 (broken), similar results occur, see Figs. 3(b1,b2). Here too, we find a stable nonlinear localized mode, while the ${\mathscr{P}}{\mathscr{T}}$ symmetry of its linear counterpart is broken, for parameters (*m*_0_, *θ*_0_) = (0.8, 0.3), see Fig. 3(b3), even if the real part of the corresponding complex potential is a weak barrier (rather than a well), whose amplitude is smaller than that of the corresponding gain-loss distribution, see Fig. 3(b4). For *θ*_0_ = 0.1, we increased *m*_0_ continuously from 0.4 to 0.6 (unbroken), so that a continuous family of stable solitons could also be readily found.

For *α* = 3, when we fix *m*_0_ = 0.2 and increase *θ*_0_ continuously from 0.1 to 0.36 (unbroken), similar results still hold, see Fig. 3(c1). On the other hand, when, for *θ*_0_ = 0.1, *m*_0_ increases to 0.54, the nonlinear localized mode may be unstable, as in Fig. 3(c2), even if the ${\mathscr{P}}{\mathscr{T}}$ symmetry of the linear state remains unbroken. Most interesting, if we increase *θ*_0_ to 0.36 in the same case, the nonlinear localized mode *restabilizes itself*, while the ${\mathscr{P}}{\mathscr{T}}$ symmetry of the linear state gets broken, see Fig. 3(c3). Another interesting feature is that the real part of the corresponding complex potential may now exhibit a double-well shape, as shown in Fig. 3(c4).

### Interactions between exact bright solitons and sech pulses

**Figure 4:**
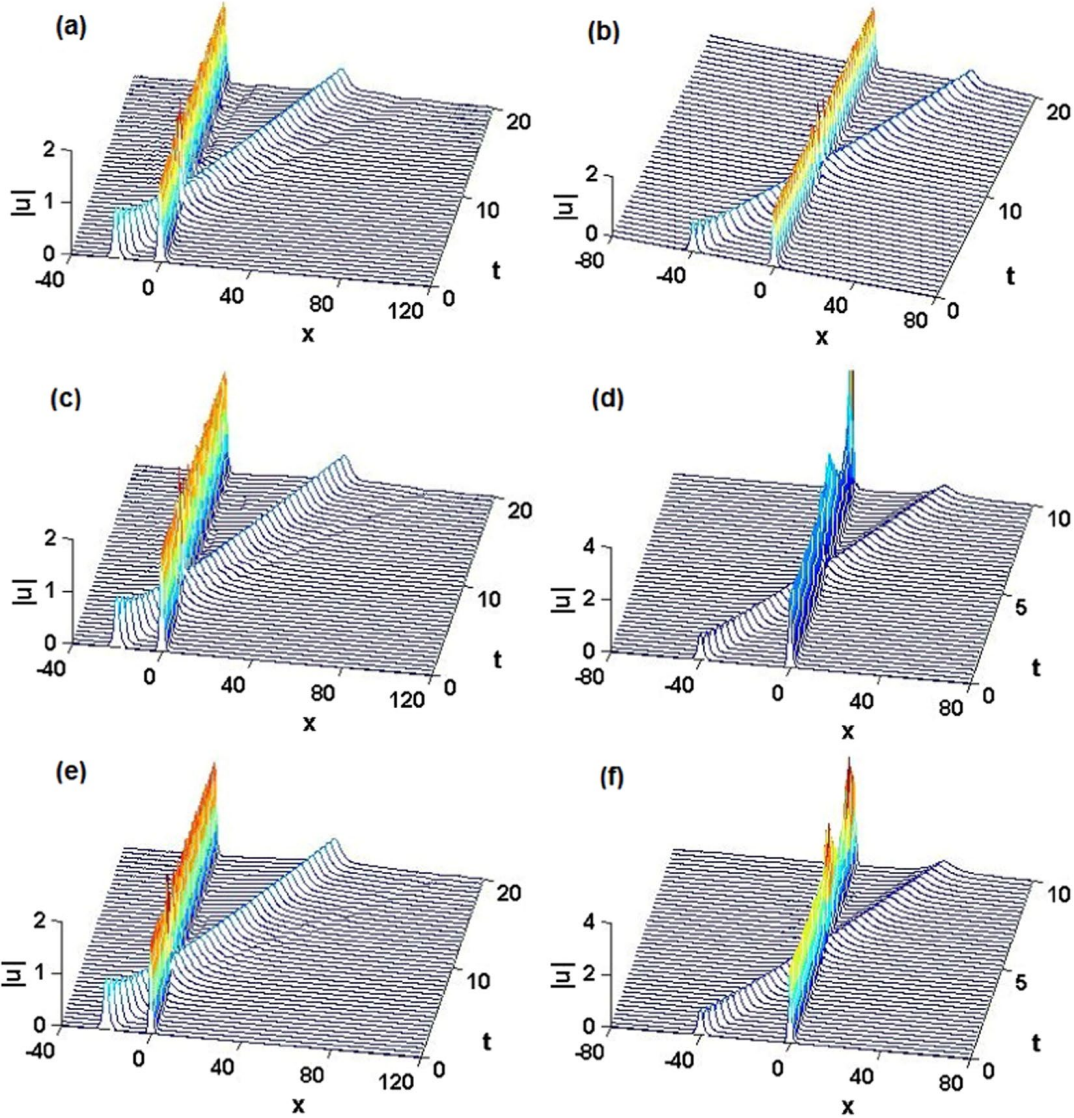
Collisions between exact bright solitons and boosted sech-shaped solitary pulses, produced by simulations of Eq. (1). (**a**) $\alpha =1,{m}_{0}=0.5,{\theta }_{0}=0.1$ (unbroken), with the input composed of the exact soliton (6) and the solitary pulse $\text{sech}(x+\mathrm{20)}{e}^{1.8ix}$, (**b**) $\alpha =1,{m}_{0}=0.75,{\theta }_{0}=0.1$ (broken), with the initial solitary pulse ${\rm sech }(x+\mathrm{40)}{e}^{2.2ix}$; (**c**) $\alpha =2,{m}_{0}=0.4,{\theta }_{0}=0.1$ (unbroken), with the initial solitary pulse $\text{sech}(x+\mathrm{20)}{e}^{1.8ix}$, (**d**) $\alpha =2,{m}_{0}=0.8,{\theta }_{0}=0.3$ (broken), with the initial solitary pulse $\text{sech}(x+\mathrm{40)}{e}^{4ix}$; (**e**) $\alpha =3,{m}_{0}=0.2,{\theta }_{0}=0.1$ (unbroken), with the initial solitary pulse $\text{sech}(x+\mathrm{20)}{e}^{1.8ix}$, (**f**) $\alpha =3,{m}_{0}=0.54,{\theta }_{0}=0.36$ (broken), with the initial solitary pulse $\text{sech}(x+\mathrm{40)}{e}^{4ix}$. Other parameters are $\alpha =1,2,3$ for the first, second, and third row, respectively. Note that t-axes should be z-axes.

To additionally test the robustness of the exact bright solitons (6), we simulated their collisions with boosted (moving) sech-shaped solitary pulses. For *α* = 1, 2, 3, we respectively choose $({m}_{0},{\theta }_{0})=\mathrm{(0.5},\mathrm{0.1)},\mathrm{(0.4},\mathrm{0.1)},\mathrm{(0.2},\mathrm{0.1)}$ with the unbroken ${\mathscr{P}}{\mathscr{T}}$ symmetry of the linear state and consider initial conditions ${\psi }_{\alpha }(x,\,\mathrm{0)}={\varphi }_{\alpha }(x)+\,{\rm sech} (x+\mathrm{20)}{e}^{1.8ix}$ with ${\varphi }_{\alpha }(x)$ given by Eq. (6), and *e* ^1.8*ix*^ imposing the boost onto the sech pulse with the unitary amplitude and initial position at *x* = −20. Direct simulations demonstrate that the exact nonlinear modes ${\varphi }_{\alpha }(x)$ propagate steadily without any change of shape and velocity after the collision, see Figs. 4(a,c,e). In other simulations, we chose values $({m}_{0},{\theta }_{0})=\mathrm{(0.75},\mathrm{0.1)},\mathrm{(0.8},\mathrm{0.3)},\mathrm{(0.54},\mathrm{0.36)}$, which correspond to the linear modes with broken ${\mathscr{P}}{\mathscr{T}}$ symmetry, and took the corresponding initial conditions as ${\psi }_{1}(x,\,\mathrm{0)}={\varphi }_{1}(x)+\,{\rm sech} (x+\mathrm{40)}{e}^{2.2ix}$ with ${\varphi }_{1}(x)$ given by Eq. (6) for *α* = 1, as well as ${\psi }_{\mathrm{2,3}}(x,\,\mathrm{0)}={\varphi }_{\mathrm{2,3}}(x)+\,{\rm sech }(x+\mathrm{40)}{e}^{4ix}$, with ${\varphi }_{\mathrm{2,3}}(x)$ given by Eq. (6) for *α* = 2, 3. In the case of *α* = 1, the exact soliton still demonstrates the steady propagation after the collision, while in the cases of *α* = 2, 3 the amplitude of the initial exact soliton rapidly grows after the collision, thus manifesting interaction-induced instability.

### Adiabatic transformation of stable nonlinear modes

Here, we elaborate three different scenario of dynamical control of nonlinear localized modes, making use of adiabatically varying parameters of the potential^38, 53–55^, *m*_0_ → *m*_0_(*z*) or *θ*_0_ → *θ*_0_(*z*). We choose the following temporal-modulation pattern:

7 $$\{{m}_{0},{\theta }_{0}\}(z)=\{\begin{array}{ll}(\{{m}_{02},{\theta }_{02}\}-\{{m}_{01},{\theta }_{01}\})\,\sin (\pi z/2000)+\{{m}_{01},{\theta }_{01}\}, & {\rm{0}}\le {\rm{z}} < \mathrm{1000},\\ \{{m}_{02},{\theta }_{02}\}, & {\rm{z}}\ge {\rm{1000}}{\rm{.}}\end{array}$$

This implies replacing Eq. (1) by

8 $$i\frac{\partial \psi }{\partial z}=[-\frac{\partial }{\partial x}m(x,z)\frac{\partial }{\partial x}+V(x,z)+iW(x,z)-g{|\psi |}^{2}]\psi ,$$

where $m(x,z),V(x,z),W(x,z)$ are given by Eqs. (2), (3) and (4) respectively, with *m*_0_ → *m*_0_(*z*) and *θ*_0_ → *θ*_0_(*z*).

**Figure 5:**
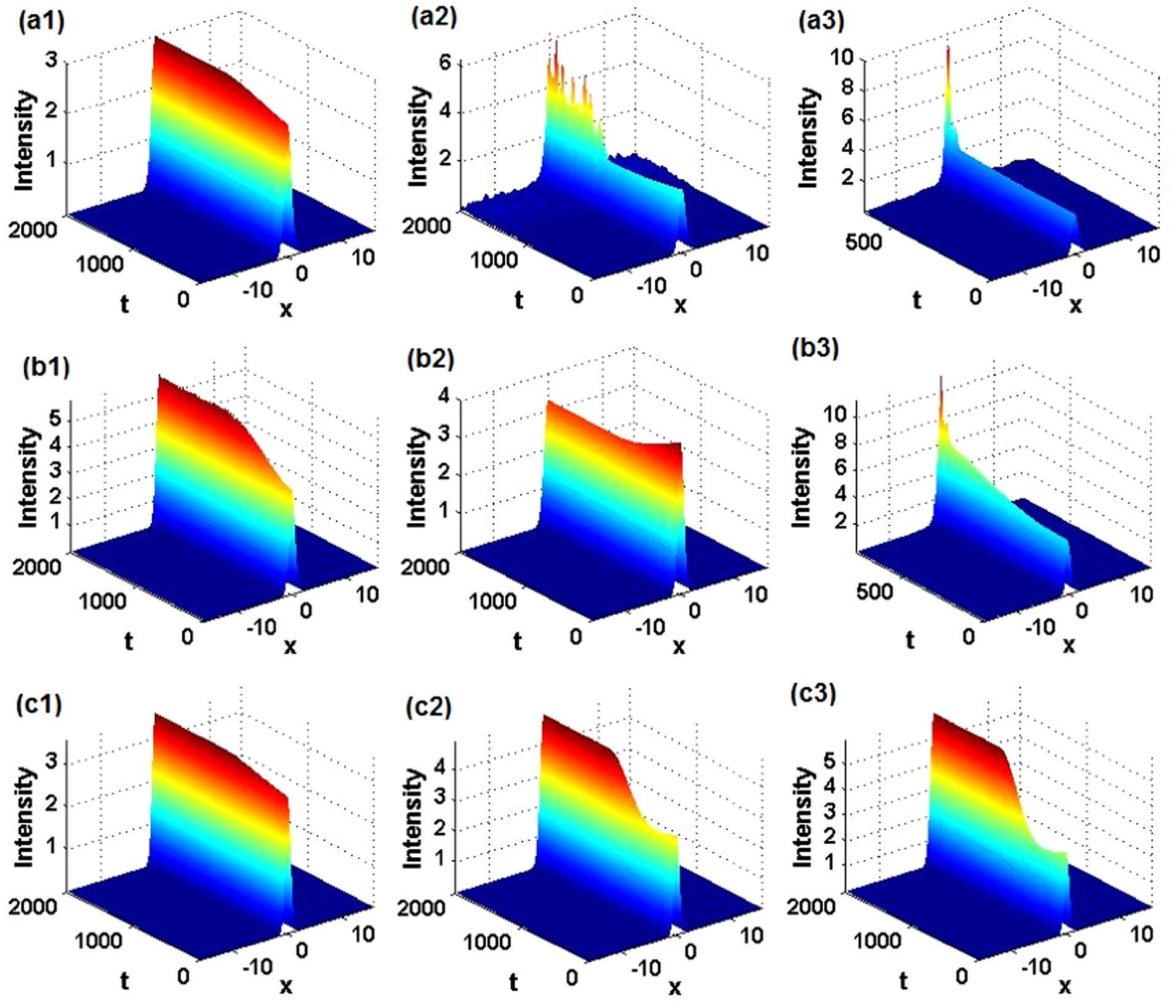
The transformation of initially stable nonlinear modes, produced by simulations of Eq. (8). (**a1**) ${m}_{01}=0.5,{\theta }_{01}=0.1,{\theta }_{02}=0.3$, (**a2**) ${m}_{01}=0.5,{m}_{02}=0.75,{\theta }_{01}=0.1$, (**a3**) ${m}_{01}=0.5,{m}_{02}=0.75,{\theta }_{01}=0.1,{\theta }_{02}=0.3$; (**b1**) ${m}_{01}=0.4,{\theta }_{01}=0.1,{\theta }_{02}=0.5$, (**b2**) ${m}_{01}=0.4,{m}_{02}=0.6,{\theta }_{01}=0.1$, (**b3**) ${m}_{01}=0.4,{m}_{02}=0.6,{\theta }_{01}=0.1,{\theta }_{02}=0.5$; (**c1**) ${m}_{01}=0.2,{\theta }_{01}=0.1,{\theta }_{02}=0.36$, (**c2**) ${m}_{01}=0.2,{m}_{02}=0.54,{\theta }_{02}=0.36$, (**c3**) ${m}_{01}=0.2,{m}_{02}=0.54,{\theta }_{01}=0.1,{\theta }_{02}=0.36$. Cases (**a1**,**b1**,**b2**,**c1**) exhibit the stable transformation between parameter sets correspond to stable linear states with unbroken ${\mathscr{P}}{\mathscr{T}}$ symmetry. (**c2**,**c3**) Stable transformation between parameter sets corresponding to unbroken and broken ${\mathscr{P}}{\mathscr{T}}$-symmetry of the linear states. (**a2**,**a3**,**b3**) Unstable transformation of the initially stable nonlinear mode between parameter sets corresponding to unbroken and broken ${\mathscr{P}}{\mathscr{T}}$-symmetry of the linear states. Other parameters are $\alpha =1,2,3$ for the first, second, and third rows, respectively. Note that t-axes should be z-axes.

For the cases of *α* = 1, 2, 3, Figs. 5(a1,b1,c1), respectively, exhibit stable switch of the nonlinear localized modes $\psi (x,z)$ governed by Eq. (8), starting from the initial condition given by Eq. (6). In these figures, we demonstrate the transformation of initially stable nonlinear localized modes for $({m}_{01},{\theta }_{01})=\mathrm{(0.5},\mathrm{0.1)},({m}_{01},{\theta }_{01})=\mathrm{(0.4},\mathrm{0.1)},({m}_{01},{\theta }_{01})=\mathrm{(0.2},\mathrm{0.1)}$, with *α* = 1, 2, 3, to ones corresponding, respectively, to $({m}_{02},{\theta }_{02})=\mathrm{(0.5},\mathrm{0.3)},({m}_{02},{\theta }_{02})=\mathrm{(0.4},\mathrm{0.5)},({m}_{02},{\theta }_{02})=\mathrm{(0.2},\mathrm{0.36)}$, i.e., *m*_0_ is fixed, while *θ*_0_ varies. In these cases, both the initial and final parameter values correspond to linear modes with unbroken ${\mathscr{P}}{\mathscr{T}}$ symmetry. Likewise, fixing *θ*_0_ and setting *m*_0_ → *m*_0_(*z*) as per by Eq. (7), we can perform similar transformations of stable exact nonlinear localized modes, as is shown in Figs. 5(a2,b2,c2). Figure 5(b2) displays a typical stable transformation of the nonlinear modes (6) with *α* = 2, also ending with parameters corresponding to the linear state with unbroken ${\mathscr{P}}{\mathscr{T}}$ symmetry.

On the other hand, Fig. 5(a2) reveals unstable transformation of the nonlinear modes (6) with *α* = 1, and Fig. 5(c2) exhibits stable transformation of the nonlinear modes (6) with *α* = 3, both from initial parameters corresponding to the linear state with unbroken ${\mathscr{P}}{\mathscr{T}}$ symmetry to final ones corresponding to broken ${\mathscr{P}}{\mathscr{T}}$ symmetry in the linear state. Finally, the transformation is also implemented by varying both *m*_0_ and *θ*_0_. Instability of such simultaneous transformation, observed in Fig. 5(a3) may be attributed to the instability of the corresponding single-parameter variation, shown above in Fig. 5(a2). Further, Fig. 5(b3) reveals that the simultaneous variation of *m*_0_ and *θ*_0_ may give rise to an unstable output, while both corresponding the single-component variations are stable, see Figs. 5(b1,b2). Nevertheless, in Fig. 5(c3) we stably transform an initially exact nonlinear mode, with unbroken ${\mathscr{P}}{\mathscr{T}}$ symmetry of the respective linear state, to another exact nonlinear mode, which corresponds to the broken ${\mathscr{P}}{\mathscr{T}}$ symmetry in the linear state, varying both parameters. The stable outcome of the simultaneous variation of *m*_0_ and *θ*_0_ may be possible if the separate variation of each parameter produced a stable output.

Thus, we conclude that the simultaneous variation of the two parameters leads to an unstable output if either of the two corresponding separate variations is unstable, see Figs. 5(a1–a3). If both separate excitations produce stable outputs, their simultaneous action may give either an unstable output, see Figs. 5(b1–b3), or a stable one, see Figs. 5(c1–c3).

### 1D constant-intensity waves

Recently, a class of complex potentials that are more general than the ${\mathscr{P}}{\mathscr{T}}$-symmetric complex-valued ones has been put forward^39, 78–80^. Similar to the ${\mathscr{P}}{\mathscr{T}}$-symmetric potentials, these more general complex potentials admit the existence of *continuous families* of stationary states, supported by the balance between gain and loss (unlike *isolated* stationary solutions found in generic dissipative systems), a part of which may be stable﻿^39, 78–80^. The real and imaginary parts of these potentials are defined^81^ *V*(*x*) = −*v* ^2^(*x*), *W*(*x*) = *dv*(*x*)/*dx*, where *v*(*x*) is an arbitrary real function. Here, we address a generalization of such potentials in the model with the variable diffraction coefficient, *m*(*x*), in the form of

9 $$V(x)=-m(x){v}^{2}(x),\,W(x)=\frac{d(m(x)v(x))}{dx},$$

where *v*(*x*) is a known real function of space. If *m*(*x*) (e.g., that defined in Eq. (2)) and *v*(*x*) are both even functions, then the complex potential given by Eq. (9) is a ${\mathscr{P}}{\mathscr{T}}$-symmetric one. In a more general case, when the potential is not ${\mathscr{P}}{\mathscr{T}}$-symmetric, it nevertheless provides for the global balance between the gain and loss, in the case of localized or periodic functions *m*(*x*)*v*(*x*), because *W*(*x*) is defined in Eq. (9) as a full derivative.

**Figure 6:**
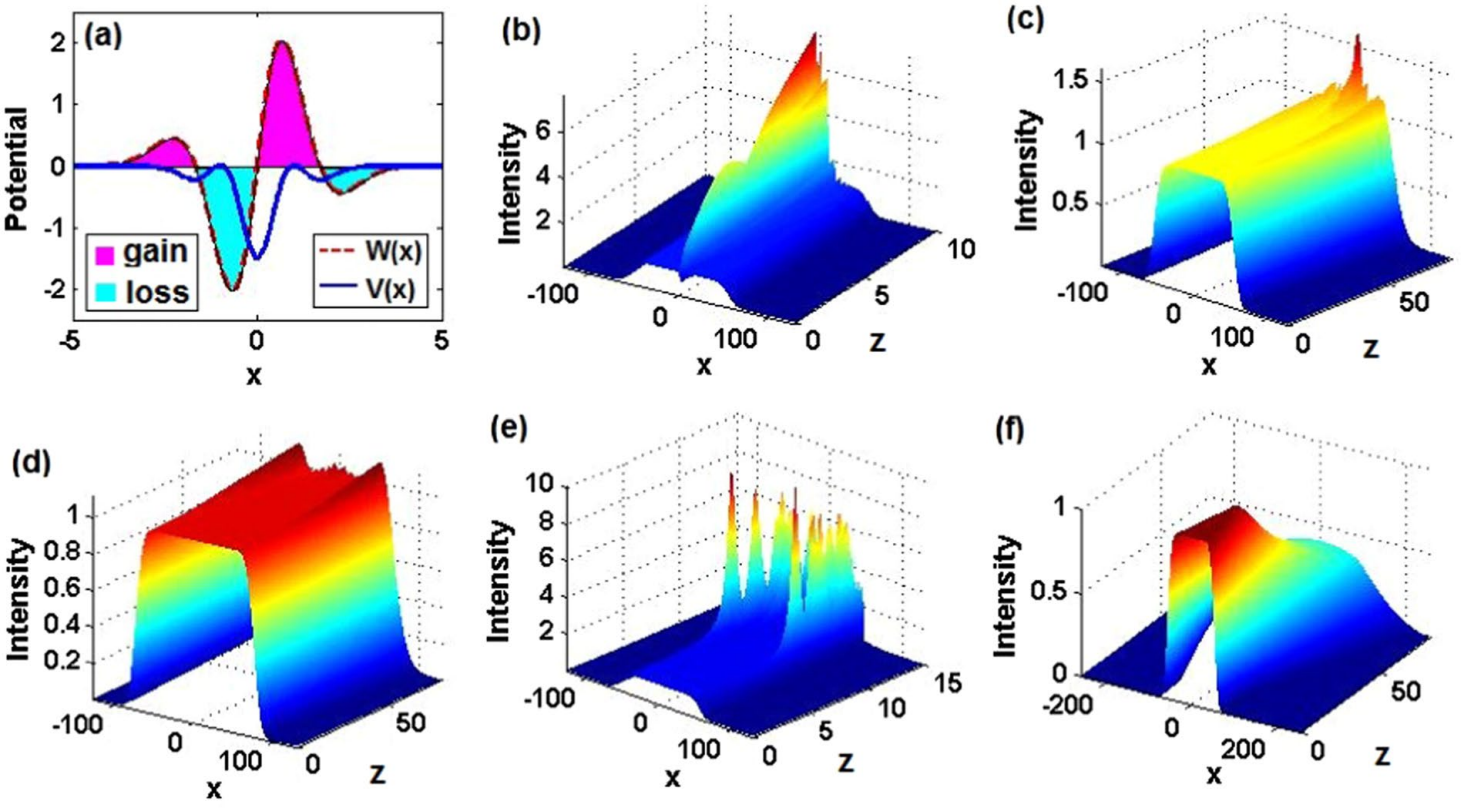
Constant-intensity waves in linear and nonlinear ${\mathscr{P}}{\mathscr{T}}$-symmetric systems with the complex potential of Hermite-Gauss (HG) type. (**a**) Real (solid blue) and imaginary (dashed red) parts of the complex potential $V(x)+iW(x)$ given by Eq. (9) hereafter, magenta- and cyan-filled areas designate the presence of the gain and loss, respectively. (**b**) The evolution of the constant-amplitude wave without the correctly introduced initial phase at *z* = 0 in the linear setting (*g* = 0). Spatial diffraction of (**c**) narrow and (**d**) wide truncated constant-intensity waves. The evolution of the constant-amplitude wave with the correct phase at *z* = 0 under (**e**) the self-focusing (*g* = 1) nonlinearity and (**f**) the self-defocusing (*g* = −1) nonlinearity.

**Figure 7:**
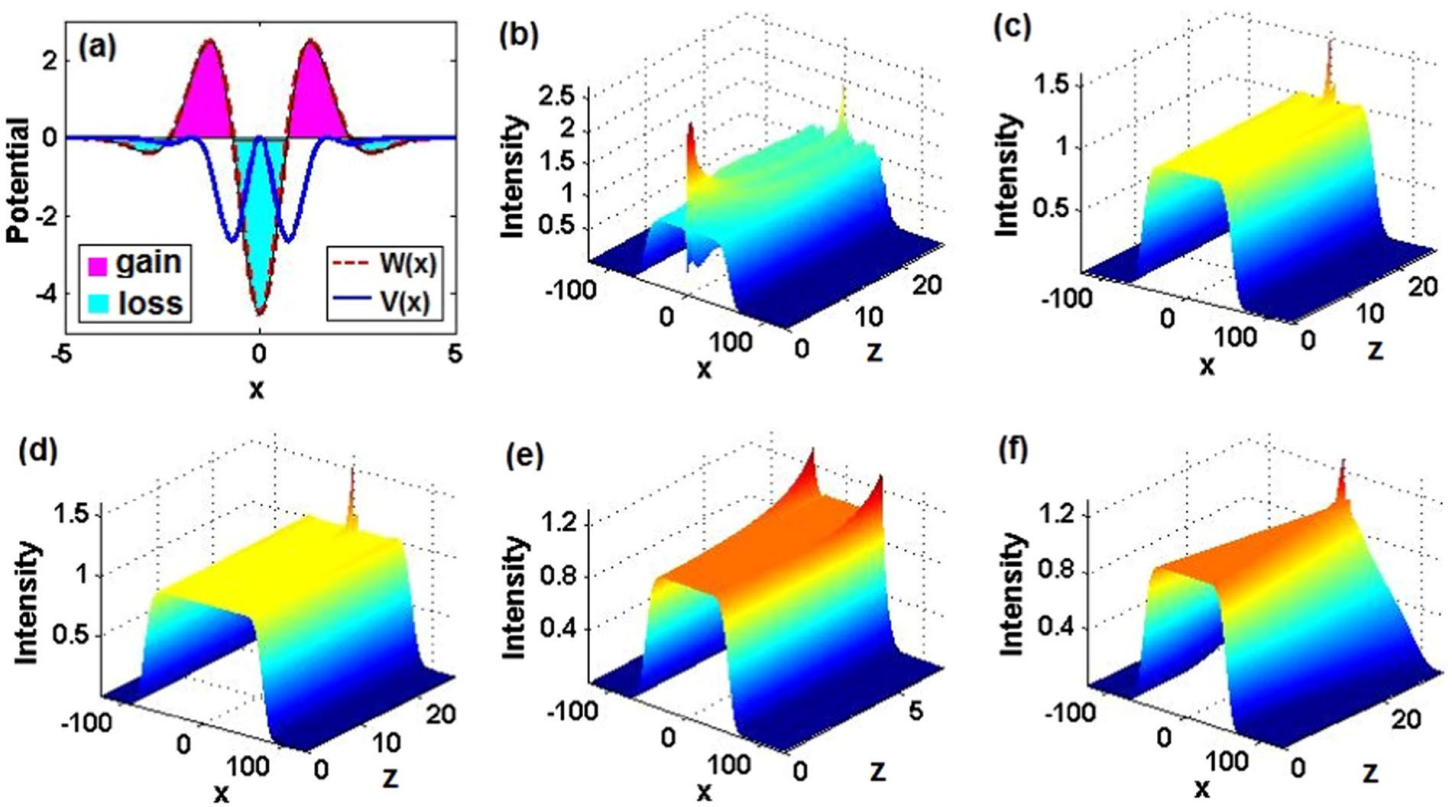
Constant-intensity waves in linear and nonlinear non-${\mathscr{P}}{\mathscr{T}}$-symmetric systems with the complex potential of HG type. The same as in Fig. 6, but for the non-${\mathscr{P}}{\mathscr{T}}$ -symmetric complex potential of the HG type, see the text.

**Figure 8:**
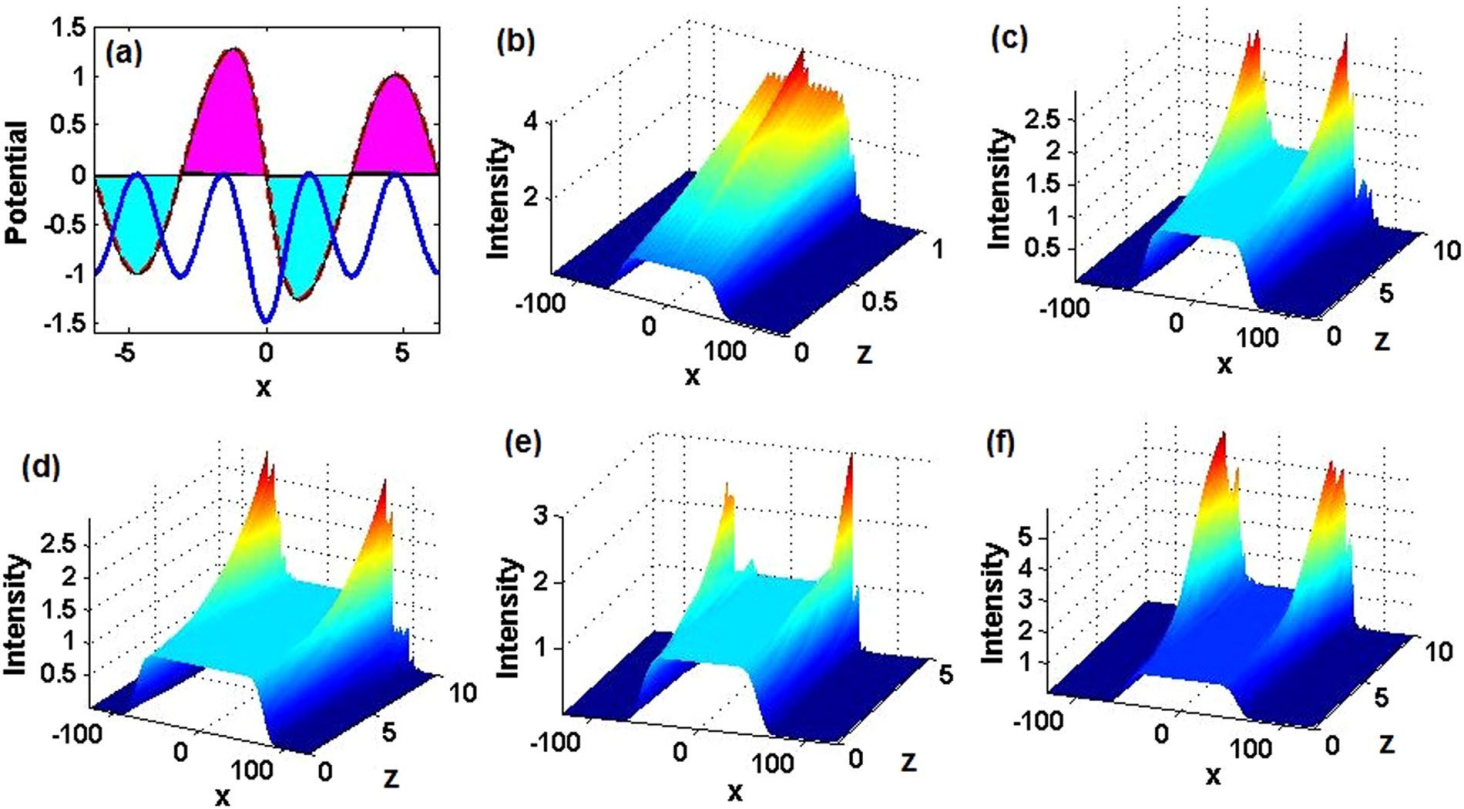
Constant-intensity waves in linear and nonlinear ${\mathscr{P}}{\mathscr{T}}$-symmetric systems with the complex potential of cos type. The same as in Figs. 6 and 7, but for the ${\mathscr{P}}{\mathscr{T}}$-symmetric complex potential of the cos type, see the text.

For the general potential taken as per Eq. (9), stationary constant-intensity (alias CW, i.e., continuous-wave) solutions of Eq. (1) with any *g* are found in the form of (see Methods)

10 $$\psi (x,z)=C\exp [i{\int }_{0}^{x}v(s)ds+ig{C}^{2}z].$$

where *C* is a real constant amplitude. The power flow (the Poynting vector) corresponding to the CW solution (10) is $S(x)={C}^{2}v(x)$. To examine dynamical properties of the CW solution (10), we consider three different types of the complex potentials (9), in which the diffraction coefficient *m*(*x*) is chosen as in Eq. (2), and *v*(*x*) is taken as an Hermite-Gauss (HG) function, ${H}_{n}(x){e}^{-\omega {x}^{2}\mathrm{/2}}$ (*H*_*n*_(*x*) is the Hermite polynomial, and $\omega  > 0$ is a frequency), or as a simple periodic one, *v*_0_ cos(*x*). Generic examples of this class of complex potentials correspond to $m(x)=0.5\,{\rm sech}(x)+1$, with $v(x)={H}_{2}(x){e}^{-{x}^{2}\mathrm{/2}}$ (a ${\mathscr{P}}{\mathscr{T}}$-symmetric HG form), or $v(x)={H}_{3}(x){e}^{-{x}^{2}\mathrm{/2}}$ (a non-${\mathscr{P}}{\mathscr{T}}$-symmetric HG form), or, finally, $v(x)=\,\cos (x)$ (a ${\mathscr{P}}{\mathscr{T}}$-symmetric periodic form). The profiles and gain-loss regions of these three complex potentials are displayed in Figs. 6a, 7(a) and 8(a), respectively. In the linear limit (*g* = 0) in these three cases, if the CW inputs are taken merely as $\psi (x,\mathrm{0)}=C$, without the correct phase given by Eq. (10), the beams grow fast in the center, leading to instability, as shown in Figs. 6(b), 7(b) and 8(b). However, if the inputs are taken as solution (10) with the correct phase, the growth of perturbations is initially suppressed, occurring later as the modulational instability of the CW (Figs. 6(c), 7(c) and 8(c)). It is worthy to note that the beam in the ${\mathscr{P}}{\mathscr{T}}$-symmetric system with the HG complex potential steadily propagates farther than that in the case of the non-${\mathscr{P}}{\mathscr{T}}$ HG complex potential, as the ${\mathscr{P}}{\mathscr{T}}$-symmetric potential naturally provides for a better balance of the gain and loss. When the truncation length of the input CW becomes larger, the stable-propagation distance increases too under the action of the ${\mathscr{P}}{\mathscr{T}}$-symmetric HG potential (see Fig. 6(d)), but it does not increase in the case of the non-${\mathscr{P}}{\mathscr{T}}$-symmetric HG potential (see Fig. 7(d)).

We have also investigated the nonlinear evolution of the CW input in the presence of the self-focusing and self-defocusing Kerr nonlinearity (*g* = +1 and −1, respectively) for the same three complex potentials. As a result, we find that the CW state maintains stable propagation over a smaller distance than that in the corresponding linear model, as can be seen in Figs. 6(e), 7(e) and 8(e) for the self-focusing nonlinearity, and in Figs. 6(f), 7(f) and 8(f) for the self-defocusing nonlinearity.

### 2D $\pmb{\mathscr{P}}\pmb{\mathscr{T}}$-symmetric nonlinear waves

Multidimensional spatial solitons are a subject of great interest to nonlinear optics^82–85^. We here consider the formation of bright spatially localized solitons in the 2D ${\mathscr{P}}{\mathscr{T}}$-symmetric setting. In this case, the field evolution is governed by the 2D NLS equation with a ${\mathscr{P}}{\mathscr{T}}$-symmetric potential:

11 $$i\frac{\partial \psi }{\partial z}=\{-\nabla [{\bf{m}}(x,y)\nabla ]+V(x,y)+iW(x,y)-g{|\psi |}^{2}\}\psi ,$$

where $\nabla$ is the 2D gradient $({\partial }_{x},{\partial }_{y})$, and **m**(*x*, *y*) is a 2 × 2 matrix function, which we take in the diagonal form, ${m}_{\alpha }(x,y)={\rm{diag}}[{m}_{\alpha }(x),{m}_{\alpha }(y)]$ with *m*_*α*_(*x*) given by Eq. (2). We look for stationary 2D modes in the form of $\psi =\varphi (x,y){e}^{i\mu z}$ with $\varphi (x,y)=u(x,y){e}^{i\theta (x,y)}$, where *u*(*x*, *y*) and *θ*(*x*, *y*) are real amplitude and phase, respectively, which satisfy stationary equations:

12 $$\begin{array}{c}{({m}_{\alpha }(x){u}_{x})}_{x}+{({m}_{\alpha }(y){u}_{y})}_{y}-[{\theta }_{x}^{2}{m}_{\alpha }(x)+{\theta }_{y}^{2}{m}_{\alpha }(y)]u-V(x,y)u-\mu u+g{u}^{3}=0,\end{array}$$

13 $${({m}_{\alpha }(x){\theta }_{x}{u}^{2})}_{x}+{({m}_{\alpha }(y){\theta }_{y}{u}^{2})}_{y}-W(x,y){u}^{2}=0.$$

The 2D ${\mathscr{P}}{\mathscr{T}}$-symmetric potentials $V(x,y)+iW(x,y)$ (i.e., *V*(*x*, *y*) = *V*(−*x*, −*y*) and *W*(−*x*, −*y*) = −*W*(*x*, *y*)), which, like in the 1D setting, admit particular exact solutions for *α* = 1, 2, and 3, are given below in Eqs. (24a), (25a) and (26a), respectively, along with the exact solutions (see Methods).

In particular, similar to ﻿the﻿ previous 1D case, it is possible to find stationary constant-intensity (CW) solutions of Eq. (11)

14 $$\psi (x,y,z)=B{e}^{i\theta (x,y)+ig{A}^{2}z},\,B={\rm{const}}$$

(cf. Eq. (10)) for a family of 2D complex potentials similar to their 1D counterparts (9):

$$V(x,y)=-\nabla \theta [(\nabla \theta ){\bf{m}}],\,W(x,y)=\nabla [(\nabla \theta ){\bf{m}}],$$

where *θ*(*x*, *y*) is an arbitrary real function. 2D CW solutions (14) will be considered in detail elsewhere.

### Comparison of exact 2D solitons and numerical solutions

**Figure 9:**
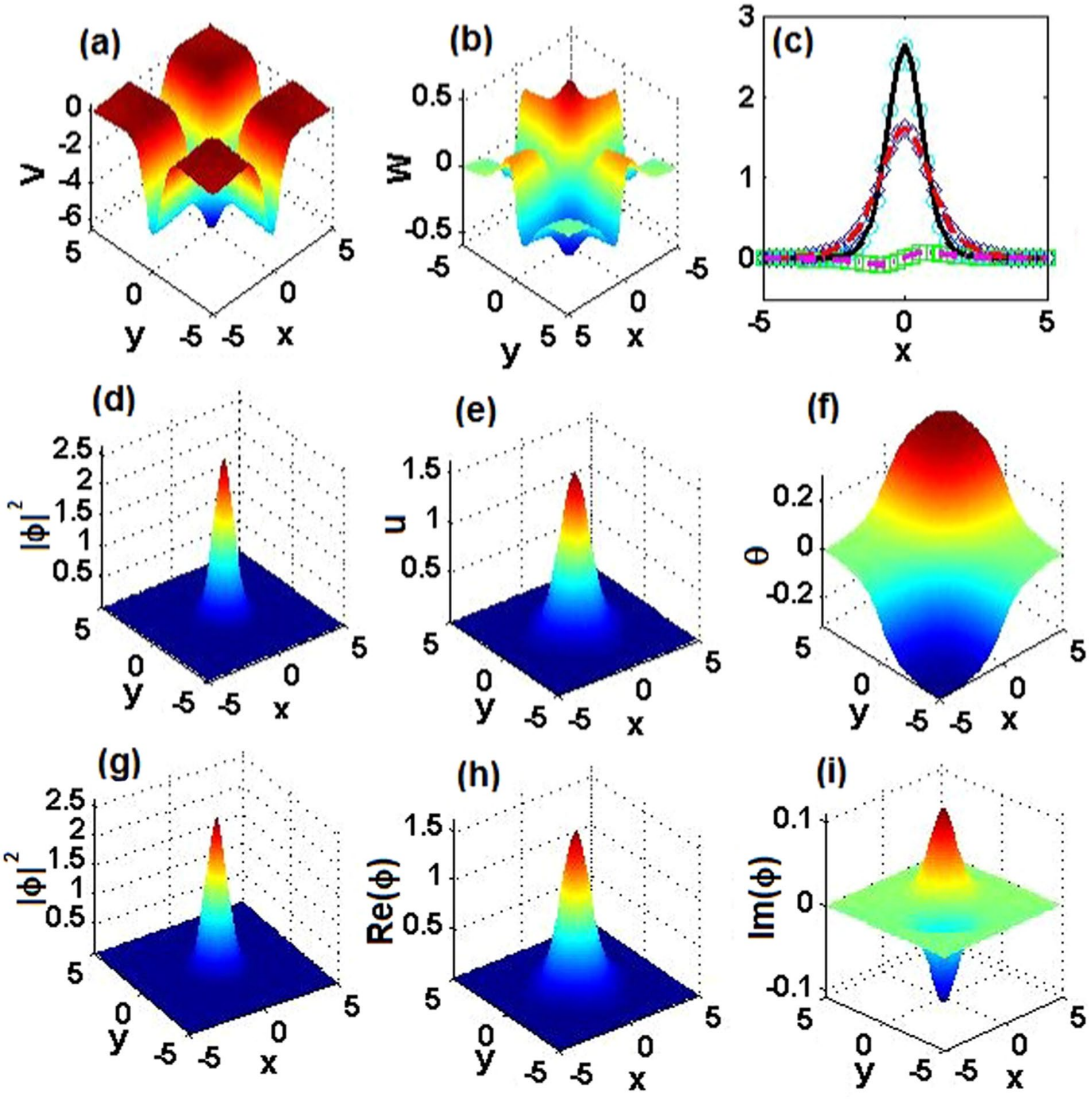
2D ${\mathscr{P}}{\mathscr{T}}$-symmetric solitons for *α* = 1. (**a**) Real and (**b**) imaginary parts of the 2D ${\mathscr{P}}{\mathscr{T}}$-symmetric complex potentials given by Eq. (24a). (**c**) Comparison of real parts, imaginary parts, and intensity of exact soliton solution ${\varphi }_{e}(x,\mathrm{0)}$ and numerically found fundamental soliton ${\varphi }_{n}(x,\mathrm{0)}$ at *μ* = 9/2, cf. Fig. 2(a1) pertaining to the 1D setting. (**d**) The intensity, (**e**) amplitude, and (**f**) phase of the exact soliton. (**g**) The intensity, (**h**) real part, and (**i**) imaginary part of the numerically found fundamental soliton. Parameters are $({m}_{0},{\theta }_{0})=\mathrm{(0.5},\mathrm{0.1)}$.

**Figure 10:**
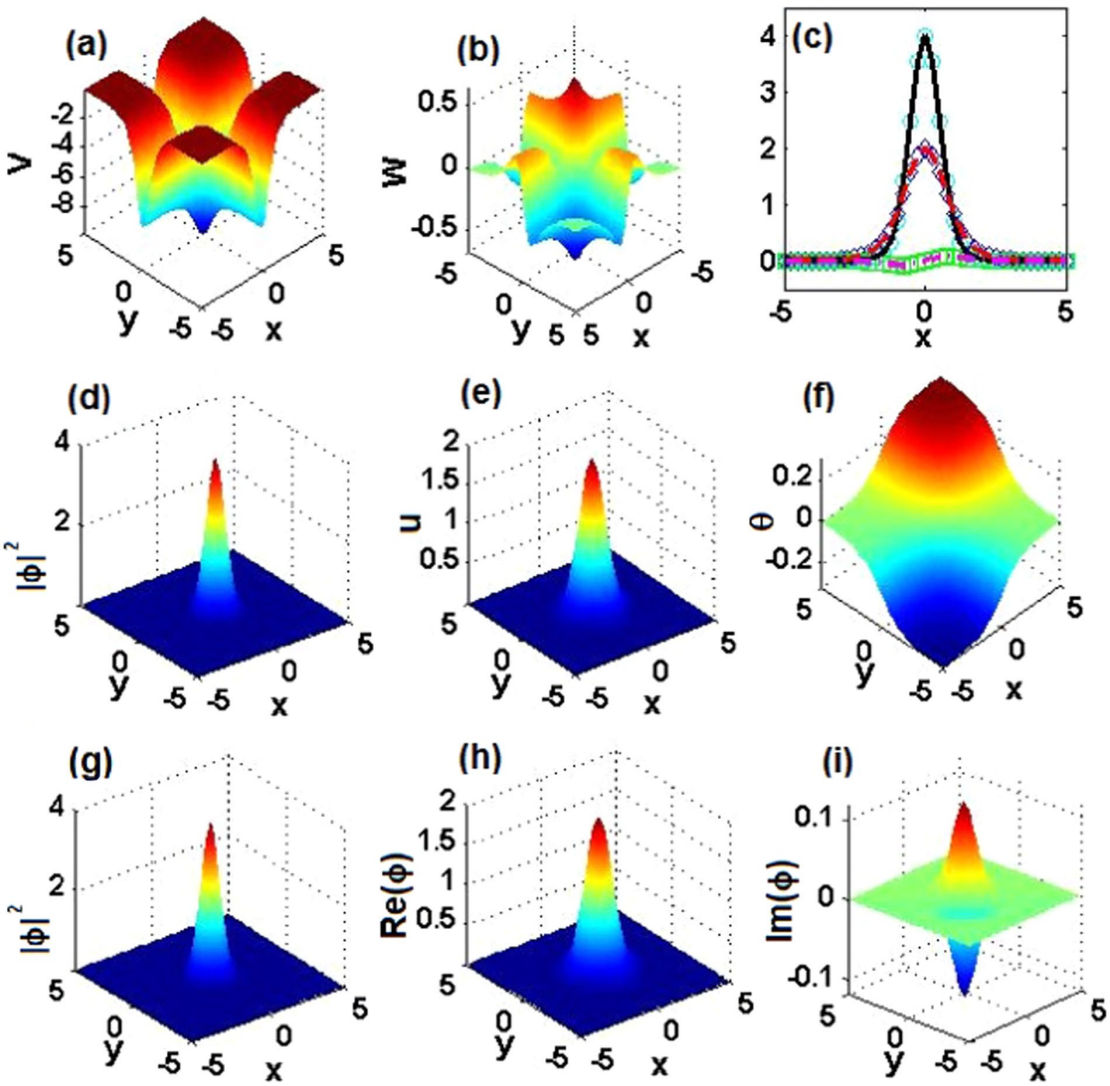
2D ${\mathscr{P}}{\mathscr{T}}$-symmetric nonlinear waves for *α* = 2. The same as in Fig. 9, but for *α* = 2, and *μ* = 8 in panel (c). Parameters are $({m}_{0},{\theta }_{0})=\mathrm{(0.4},\mathrm{0.1)}$.

**Figure 11:**
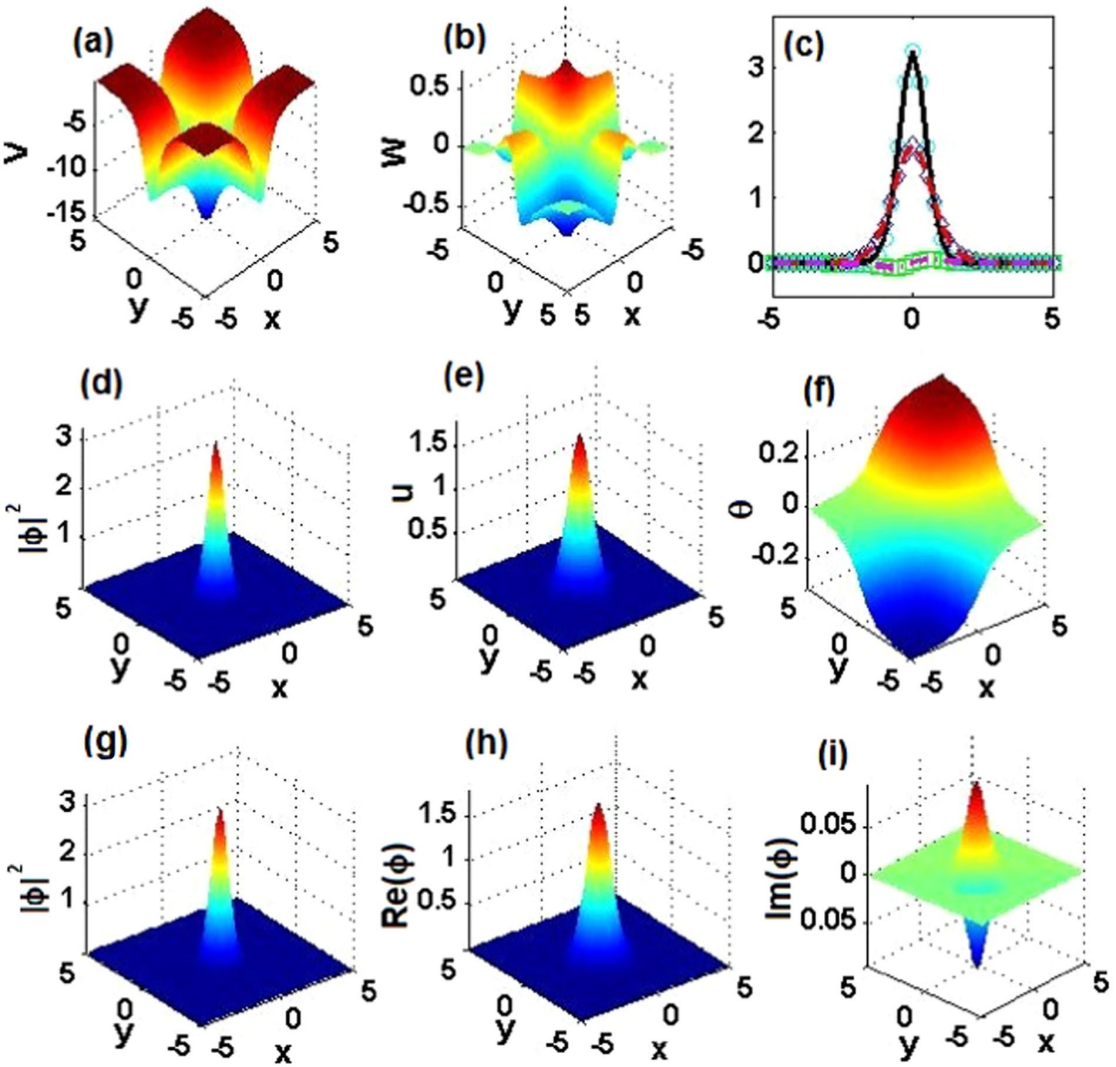
2D ${\mathscr{P}}{\mathscr{T}}$-symmetric nonlinear waves for *α* = 3. The same as in Fig. 9, but for *α* = 3, and *μ* = 25/2 in panel (c). Parameters are $({m}_{0},{\theta }_{0})=\mathrm{(0.2},\mathrm{0.1)}$.

For *α* = 1, 2, 3, we first choose three parameter sets, $({m}_{0},{\theta }_{0})=\mathrm{(0.5},\mathrm{0.1)},\mathrm{(0.4},\mathrm{0.1)},\mathrm{(0.2},\mathrm{0.1)}$, with the corresponding 2D ${\mathscr{P}}{\mathscr{T}}$-symmetric complex potentials shown in Figs. 9(a,b), 10(a,b) and 11(a,b), respectively. The corresponding exact soliton solutions, given by Eqs. (24a), (25a) and (26a), with *μ* = 9/2, 8, and 25/2, are shown in the second row of Figs. 9, 10 and 11, respectively.

To verify the analytical soliton solutions, we have numerically found the corresponding stationary fundamental solitons of Eq. (11), which are displayed in the third row of Figs. 9, 10 and 11, respectively. As is shown in Figs. 9(c), 10(c) and 11(c), the difference between the exact solutions and their numerical counterparts falls below 10^−9^, hence the exact solutions are correct.

**Figure 12:**
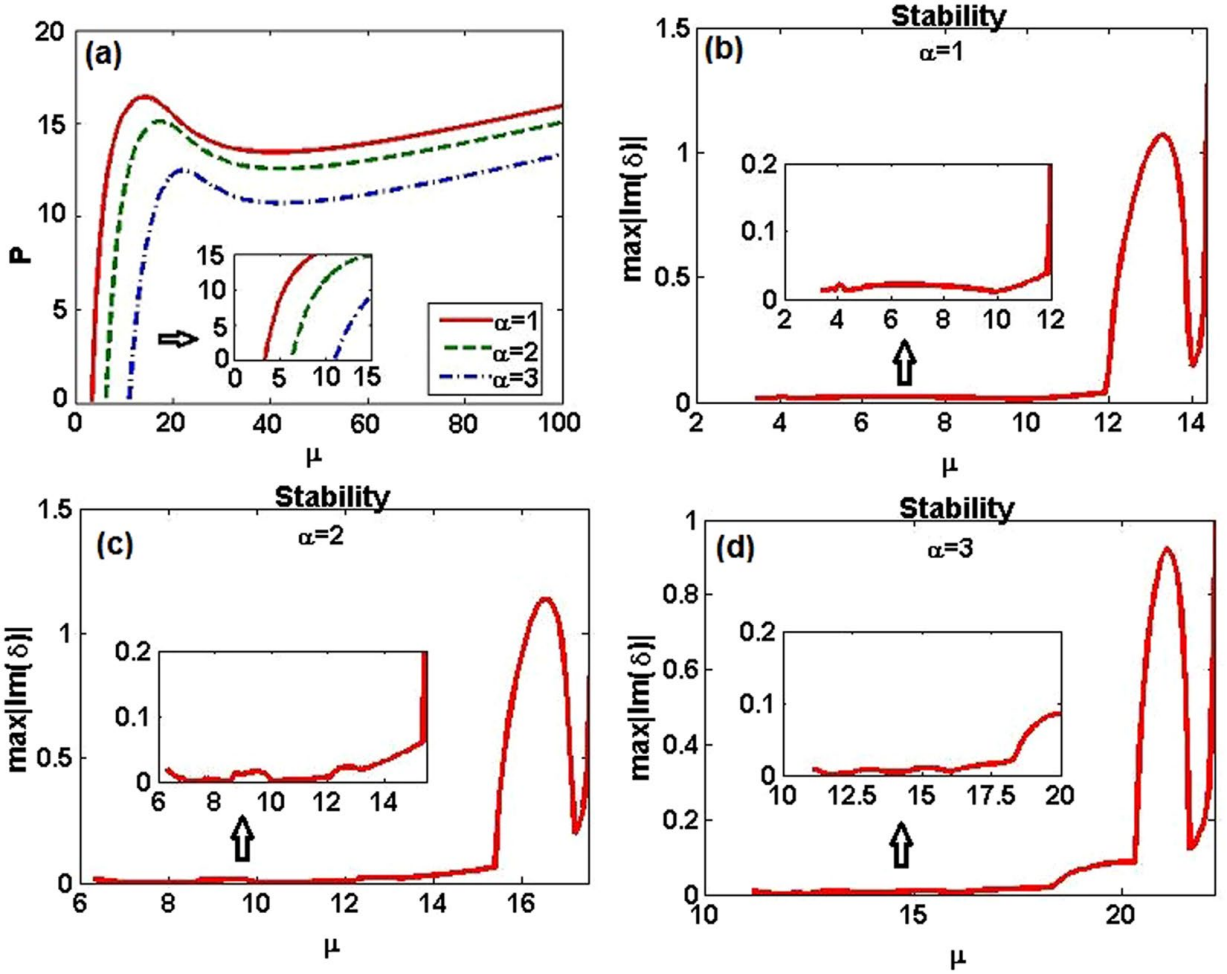
2D integral power *P* and linear stability versus the soliton’s propagation constant, −*μ*. (**a**) Power *P* versus *μ*. For the three different cases, $(\alpha ,{m}_{0},{\theta }_{0})=\mathrm{(1},0.5,\mathrm{0.1)},\mathrm{(2},0.4,\mathrm{0.1)},\mathrm{(3},0.2,\mathrm{0.1)}$, there are no upper cutoffs of the existence domains for the solitons. The corresponding lower cutoffs are ${\mu }_{1}\approx 3.4,{\mu }_{2}\approx 6.3,{\mu }_{3}\approx 11.1$, respectively. (**b**–**d**) The linear-stability spectra of numerically found fundamental solitons versus *μ*, around the corresponding exact nonlinear modes at *μ* = 9/2, 8, 25/2, respectively. The insets in (**b**–**d**) clearly show a portion of stability results when *μ* is located in lower and smaller intervals.

Furthermore, using the numerical method, we calculate the integral power at other values of the soliton parameter *μ* and identify the existence ranges of the numerically found solitons, as shown in Fig. 12(a), where the lower cutoffs are ${\mu }_{1}\approx 3.4$, ${\mu }_{2}\approx 6.3$, ${\mu }_{3}\approx 11.1$, and there are no finite upper cutoffs. These numerical soliton solutions are more often than not unstable, especially for larger values of *μ*. We display their linear-stability spectra in Figs. 12(b–d) around *μ* = 9/2, 8, and 25/2, respectively, i.e., around the values at which the corresponding exact nonlinear modes exist. As a result, we find that in first case, $(\alpha ,{m}_{0},{\theta }_{0})=\mathrm{(1},0.5,\mathrm{0.1)}$, almost all the corresponding numerical and exact solitons are unstable; however, in the second and third cases, *viz*., $(\alpha ,{m}_{0},{\theta }_{0})=\mathrm{(2},0.4,\mathrm{0.1)}$ and (3, 0.2, 0.1), there exist some stability regions, which are distributed around *μ* = 8 and 25/2. To confirm these linear-stability results, in what follows we investigate the dynamics by dint of direct numerical simulations.

### Dynamical behavior of 2D nonlinear modes

**Figure 13:**
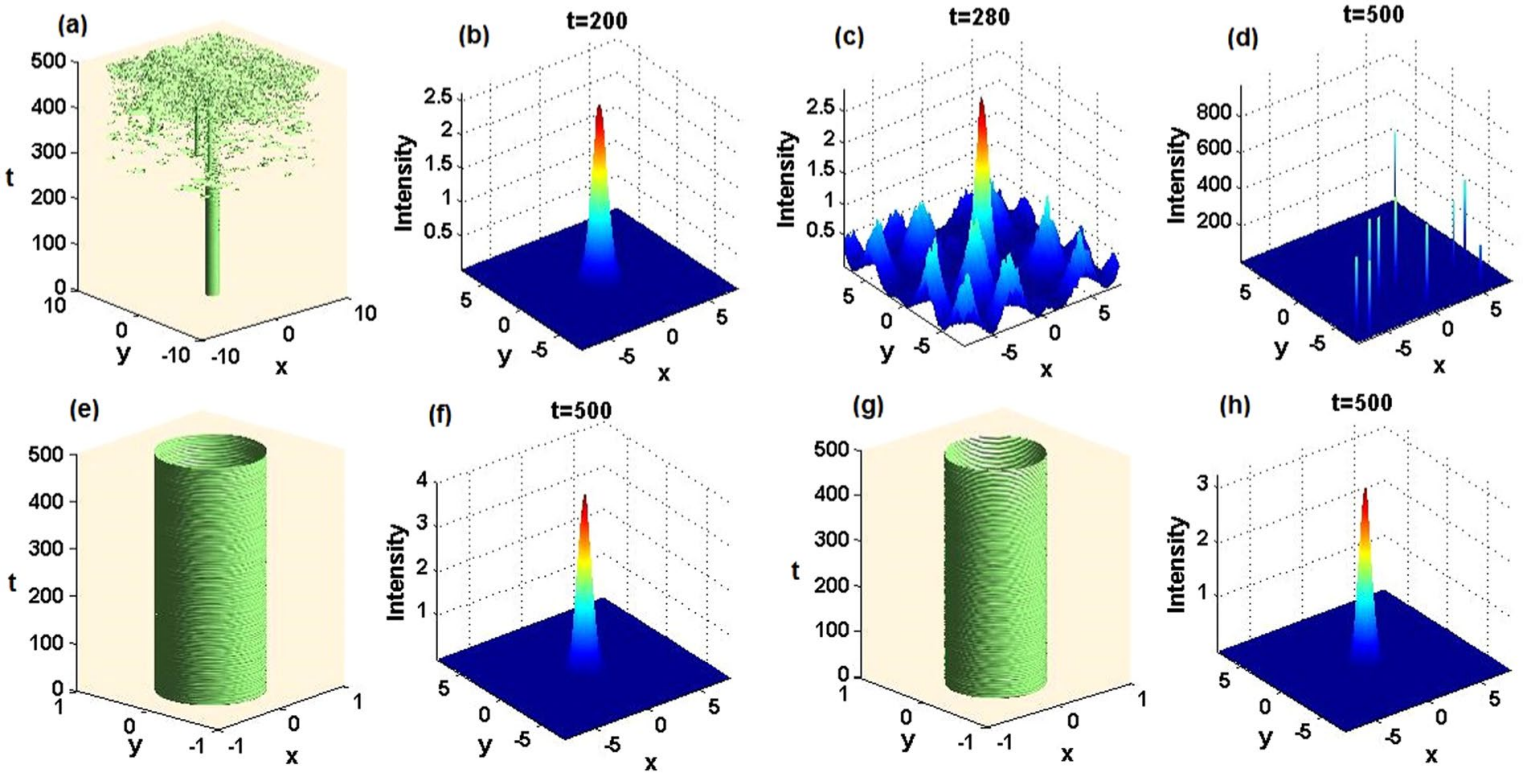
Stable and unstable evolution of 2D solitons. (**a**) The unstable evolution in terms of the intensity isosurfaces, corresponding to half the maximum initial intensity, of the 2D soliton from Fig. 9 (these and other results are virtually identical for exact solitons and their numerically found counterparts); (**b**) apparently stable and (**c**) intermediate states with emergent instability states; (**d**) the unstable final state. (**e**) The stable evolution of the soliton from Fig. 10, and (**f**) its final shape. (**g**) The stable evolution of the soliton from Fig. 11, and (**h**) its final shape. Note that t-axes ﻿should be z-axes in (**a**,**e**) and “t=” sh﻿ould be “z=” in (**b**,**c**,**d**,**f**,**g**,**h**).

To display the evolution of the 2D exact or numerically found nonlinear modes, we plot the corresponding intensity isosurfaces. For *α* = 1, as shown in Figs﻿. 13(a,d), instability is produced by simulations of the long-time evolution, although at shorter times, such as *t* = 200, the solution seems as a stable one, see Fig. 13(b). The instability sets in at $t\approx 280$ (Fig. 13(c)). However, for *α* = 2 and 3, both Figs﻿. 13(e,g) exhibit fully robust evolution of the initial-state solitons taken from Figs. 10 and 11, respectively. To confirm their stability, we display the corresponding final-state soliton profiles in Figs. 13(f,h), which are identical to the corresponding initial profiles in Figs. 10 and 11.

**Figure 14:**
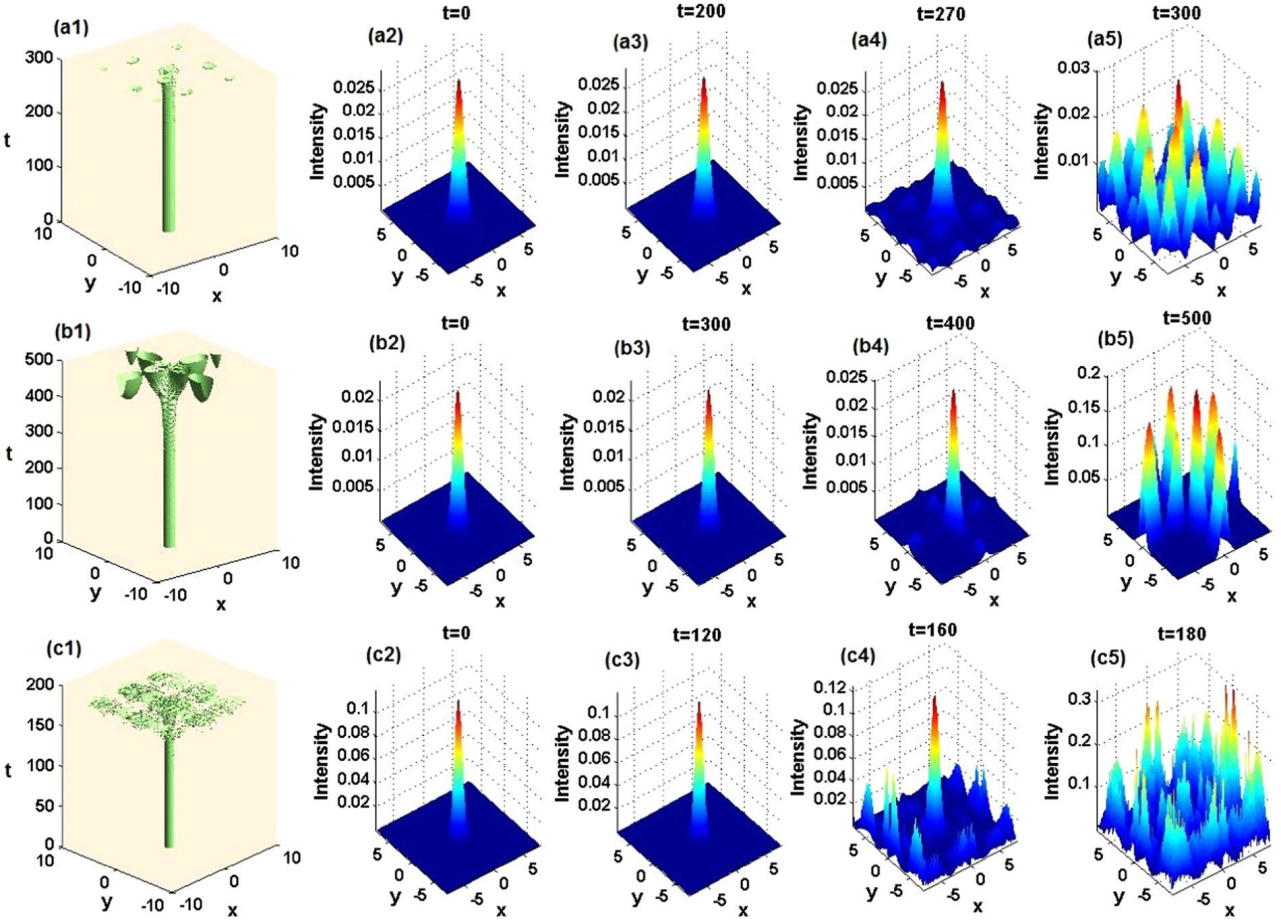
Unstable evolution of 2D solitons. (**a1**,**b1**,**c1**) The unstable evolution of numerical found solitons corresponding to ${\mu }_{1}=3.4,{\mu }_{2}=6.3,{\mu }_{3}=11.1$ for $\alpha =1,2,3$, respectively. (**a2**,**b2**,**c2**) The corresponding initial states; (**a3**,**b3**,**c3**) apparently stable intermediate states; (**a4**,**b4**,**c4**) becoming-unstable intermediate states; (**a5**,**b5**,**c5**) unstable final states. Note that t-axes should be z-axes in (**a1**,**b1**,**c1**) and “t=” should be “z=” in (**a2**,**a3**,**a4**,**a5**,**b2**,**b3**,**b4**,**b5**,**c2**,**c3**,**c4**,**c5**).

We have also numerically investigated the stability of the solitons at the lowest-power points *μ*_1_ = 3.4, *μ*_2_ = 6.3, and *μ*_3_ = 11.1 for *α* = 1, 2, and 3, respectively. They all turn out to be unstable, although they may seem stable at a shorter propagation distance. Figure 14 specifically exhibits their evolution and the corresponding intermediate and final states. These results indicate a somewhat surprising fact that the localized nonlinear mode with the lowest power is not necessarily stable. On the other hand systematic simulations demonstrate that the numerically found solitons are fully stable in the vicinity of the corresponding exact nonlinear modes, that is, near ${\mu }_{1}=\mathrm{9/2},{\mu }_{2}=8,{\mu }_{3}=\mathrm{25/2}$ for *α* = 1, 2, 3. For this reason, the exact soliton solutions are especially important ones, as they help to spot stability areas for broad soliton families.

It is worthy to note that the dynamical-stability results are in good agreement with the predictions produced above by the linear-stability analysis (see Figs. 12(b–d)). Thus, the latter analysis, in the combination with systematic direct simulations, make it possible to identify soliton-stability regions in a reliable form.

## Discussion

We have reported analytical and numerical results for new classes of 1D and 2D stable spatial solitons in cubic nonlinear media with the ${\mathscr{P}}{\mathscr{T}}$-symmetric generalized Scarf-II potentials and variable (position dependent) diffraction coefficients. First, in the linear version of the model, parameter regions of the unbroken and broken ${\mathscr{P}}{\mathscr{T}}$ symmetry have been numerically delineated. Then, in the presence of the Kerr nonlinearity, particular exact solutions for nonlinear localized modes with real eigenvalues have been obtained in the analytical form, and verified numerically. These solitons are shown to be stable through the linear-stability analysis and by means of direct simulations, in wide ranges of the governing parameters. It is worthy to note that the addition of the Kerr nonlinearity can *fix* the broken ${\mathscr{P}}{\mathscr{T}}$ symmetry of the linear system, transforming complex eigenvalues into real ones. In addition, stable bright solitons have been found in parameter regions where the ${\mathscr{P}}{\mathscr{T}}$ symmetry of the linear states is broken, for various shapes of the underlying real part of the potential, such as single- and double-well forms. Finally, interactions and adiabatic transformations of the exact solitons have been studied in detail, and the existence range and propagation dynamics of numerically found solitons have been examined too. We also study the evolution of constant-intensity waves in a model combining the variable diffraction coefficient and complex potentials with globally balanced gain and loss, which are more general than ${\mathscr{P}}{\mathscr{T}}$-symmetric ones, but feature similar properties. These theoretical results suggest new experiments for ${\mathscr{P}}{\mathscr{T}}$-symmetric nonlinear waves in nonlinear and nonuniform optical media, and provide useful theoretical guidance for studies in related fields, such as BECs.

## Methods

### Nonlinear stationary modes

Stationary solution of Eq. (1) are looked for in the usual form, $\psi (x,z)=\varphi (x){e}^{i\mu z}$, where −*μ* is the propagation constant, and the localized complex wave function satisfies the following ordinary differential equation with the *x*-dependent diffraction coefficient:

15 $$[\frac{d}{dx}m(x)\frac{d}{dx}-V(x)-iW(x)+g{|\varphi |}^{2}]\varphi =\mu \varphi .$$

We take the complex wave function as $\varphi (x)=u(x)\,\exp (i{\int }_{0}^{x}v(s)ds)$ with real amplitude *u*(*x*), and superfluid phase velocity given by

16 $$v(x)=\frac{1}{m(x){u}^{2}(x)}{\int }_{0}^{x}W(s){u}^{2}(s)ds.$$

The amplitude satisfies the following second-order ordinary differential equation:

17 $$\frac{d}{dx}[m(x)\frac{du}{dx}]=[V(x)+m(x){v}^{2}(x)-g{u}^{2}+\mu ]u,$$

which may be transformed by setting $\tilde{u}(x)\equiv m(x){u}_{x}$ into a system of coupled first-order equations:

18 $$\frac{du(x)}{dx}=\frac{\tilde{u}(x)}{m(x)},$$

19 $$\frac{d\tilde{u}(x)}{dx}=[V(x)+m(x){v}^{2}(x)-g{u}^{2}+\mu ]u,$$

solvable as a boundary-value problem by means of standard shooting methods^60^. To achieve a higher precision and computation speed, we actually used the spectral renormalization method^86^ with some necessary modifications. The method is spectrally efficient and relatively easy to implement not only in the 1D case but also in the higher-dimensional settings.

### Implementation of the numerical solution

To construct 1D localized solutions, one first needs to develop a convergent iteration, to guarantee that the amplitude neither blows up nor decays to zero. This may be realized by setting $\varphi (x)=\lambda w(x)$, where *λ* is a constant to be determined. Using the Fourier transform and the modified spectral renormalization method^86^, we thus arrive as the following iteration scheme:

20 $$\hat{w}=\frac{1}{{k}_{x}^{2}+p}{F}_{1}+\frac{{\lambda }^{2}}{{k}_{x}^{2}+p}{F}_{2},$$

where ${F}_{1}= {\mathscr F} \{[{m}_{x}{ {\mathscr F} }^{-1}(i{k}_{x}\hat{w})-(V+iW)w-\mu w+pmw]/m\}$, ${F}_{2}= {\mathscr F} (g{|w|}^{2}w/m)$, ${\lambda }^{2}={\int }_{-\infty }^{+\infty }[({k}_{x}^{2}+p){|\hat{w}|}^{2}-{F}_{1}{\hat{w}}^{\ast }]\,{\rm{d}}{k}_{x}/{\int }_{-\infty }^{+\infty }{F}_{2}{\hat{w}}^{\ast }{\rm{d}}{k}_{x}$, ${\mathscr F}$ denotes the 1D Fourier transform, and *p* is an appropriate positive constant (here *p* = 10 is taken for the 1D case). A Gaussian or sech can be taken as an input, which eventually leads to absolute errors <10^−9^, in both the convergence criterion and the numerically obtained solution satisfying Eq. (17). In general, one may restrict the number of iterations to <100; however, for the sake of high precision, we admitted up to 1000 iterative steps. Once the above-mentioned conditions for two absolute errors are satisfied simultaneously, the desired numerical soliton is obtained as $\varphi (x)=\lambda { {\mathscr F} }^{-1}[\hat{w}(x)]$.

In the 2D case, we needed to accordingly change *F*_1_ in Eq. (20), although the iteration scheme ran similar to its counterpart in the 1D case:

21 $$\hat{w}=\frac{1}{{k}_{x}^{2}+{k}_{y}^{2}+p}{F}_{1}+\frac{{\lambda }^{2}}{{k}_{x}^{2}+{k}_{y}^{2}+p}{F}_{2},$$

where ${F}_{1}= {\mathscr F} \{[m(x)-1]\,{ {\mathscr F} }^{-1}(-{k}_{x}^{2}\hat{w})+[m(y)-1]{ {\mathscr F} }^{-1}(-{k}_{y}^{2}\hat{w})+{m}_{x}(x)\,{ {\mathscr F} }^{-1}(i{k}_{x}\hat{w})$ +${m}_{y}(y)\,{ {\mathscr F} }^{-1}(i{k}_{y}\hat{w})$ $-(V+iW)\,w-\mu w+pw\}$, ${\mathscr F}$ denotes, the 2D Fourier transformation, and *p* is an appropriate positive constant (here *p* = 100 is taken for the 2D case). Other settings and procedures are similar to those in the 1D case.

### The linear-stability analysis

For the given position-dependent function *m*(*x*) and complex-valued ${\mathscr{P}}{\mathscr{T}}$-symmetric potential *V*(*x*) + *iW*(*x*), one may solve Eq. (17) (or equivalently, Eqs. (18) and (19)), to obtain stationary soliton solutions $\varphi (x)$, by analytical or the above-mentioned methods. Then localized nonlinear modes of Eq. (1) can be found in the stationary form, as $\psi (x,z)=\varphi (x){e}^{i\mu z}$. To explore the linear stability of the localized modes in the 1D case, we consider a perturbed solution^87, 88^,

22 $$\psi (x,z)=\{\varphi (x)+\epsilon [F(x){e}^{i\delta z}+{G}^{\ast }(x){e}^{-i{\delta }^{\ast }z}]\}{e}^{i\mu z},$$

where $\epsilon$ is an infinitesimal perturbation amplitude, *F*(*x*) and *G*(*x*) are the eigenfunctions of the linearized problem, and −*δ* is the respective eigenfrequency, the instability taking place if some eigenvalues are not purely real. Inserting the perturbed solution (22) into Eq. (1) and linearizing with respect to $\epsilon$, we obtain the following eigenvalue problem:

23 $$(\begin{array}{cc}{\hat{L}}_{1} & g{\varphi }^{2}\\ -g{\varphi }^{\ast 2} & -{\hat{L}}_{1}^{\ast }\end{array})\,(\begin{array}{c}F(x)\\ G(x)\end{array})=\delta (\begin{array}{c}F(x)\\ G(x)\end{array}),$$

where ${\hat{L}}_{1}\equiv {\partial }_{x}(m(x){\partial }_{x})-[V(x)+iW(x)]+2g{|\phi |}^{2}-\mu$. The ${\mathscr{P}}{\mathscr{T}}$-symmetric nonlinear modes are linearly stable provided that *δ* has no imaginary part, otherwise they are linearly unstable. The whole stability spectrum *δ* can be numerically calculated by the Fourier collocation method (see ref. 88).

For the 2D case (11), the operator ${\hat{L}}_{1}$ in Eq. (23) is changed into ${\hat{L}}_{1}={\partial }_{x}[m(x){\partial }_{x}]+{\partial }_{y}[m(y){\partial }_{y}]-[V(x,y)+iW(x,y)]+2g{|\varphi |}^{2}-\mu$. Other technical details are similar to those in the 1D case. As concerns the numerical computation of the full stability spectrum in the 2D case, the Fourier-collocation method is usually of low precision for a small number of Fourier modes. If one increases the number of the modes for a higher accuracy, the necessary size of the dense matrix corresponding to the eigenvalue problem may become prohibitively large^88^. Therefore the full 2D linear-stability spectrum in the (*m*_0_, *θ*_0_) space is not displayed any more, because of the necessary large space size and number of Fourier modes. But we will roughly depict the 2D linear-stability spectrum with respect to the soliton parameter *μ* (see Figs. 12(b–d)), in contrast to the dynamical stability of long-time wave propagation. By the way, on the account of the same reason that large spatial domains and number of the Fourier modes are necessary for a high accuracy in the 2D case, the corresponding ${\mathscr{P}}{\mathscr{T}}$-symmetric linear spectra are not exhibited further. Nevertheless, through repeated numerical tests, we find that it is instructive that the ${\mathscr{P}}{\mathscr{T}}$-symmetric breaking curves in the 1D case can provide powerful reference for those in the 2D case.

### 2D nonlinear modes for the ${\mathscr{P}}{\mathscr{T}}$-symmetric potentials with different parameters *α*

The 2D ${\mathscr{P}}{\mathscr{T}}$-symmetric potentials and exact solutions of Eqs. (12) and (13), which they admit, are listed as follows:

*Case 1* (*α* = 1):

24a $$\begin{array}{ccc}{{\bf{m}}}_{1}(x,y) & = & {\rm{d}}{\rm{i}}{\rm{a}}{\rm{g}}({m}_{0}{\rm{s}}{\rm{e}}{\rm{c}}{\rm{h}}x+1,{m}_{0}{\rm{s}}{\rm{e}}{\rm{c}}{\rm{h}}y+1),\\ {V}_{1}(x,y) & = & \frac{1}{4}\sum _{\sigma =x,y}[15{m}_{0}{\rm{s}}{\rm{e}}{\rm{c}}{\rm{h}}\sigma -(4{\theta }_{0}^{2}+15){{\rm{s}}{\rm{e}}{\rm{c}}{\rm{h}}}^{2}\sigma -{m}_{0}(4{\theta }_{0}^{2}+21)\\  &  & \times \,{{\rm{s}}{\rm{e}}{\rm{c}}{\rm{h}}}^{3}\sigma ]+{m}_{0}({\theta }_{0}^{2}+\frac{21}{4}){({\rm{s}}{\rm{e}}{\rm{c}}{\rm{h}}x{\rm{s}}{\rm{e}}{\rm{c}}{\rm{h}}y)}^{3},\\ {W}_{1}(x,y) & = & -{\theta }_{0}\sum _{\sigma =x,y}{\rm{s}}{\rm{e}}{\rm{c}}{\rm{h}}\sigma \tanh \sigma (5{m}_{0}{\rm{s}}{\rm{e}}{\rm{c}}{\rm{h}}\sigma +4),\\ {\varphi }_{1}(x,y) & = & \sqrt{{m}_{0}(4{\theta }_{0}^{2}+21)/(4g)}{({\rm{s}}{\rm{e}}{\rm{c}}{\rm{h}}x{\rm{s}}{\rm{e}}{\rm{c}}{\rm{h}}y)}^{3/2}\\  &  & \times \,\exp \{i{\theta }_{0}[{\tan }^{-1}(\sinh x)+{\tan }^{-1}(\sinh y)]\},\end{array}$$

where *μ* = 9/2.

*Case 2* (*α* = 2):

25a $$\begin{array}{ccc}{{\bf{m}}}_{2}(x,y) & = & {\rm{d}}{\rm{i}}{\rm{a}}{\rm{g}}({m}_{0}{{\rm{s}}{\rm{e}}{\rm{c}}{\rm{h}}}^{2}x+1,{m}_{0}{{\rm{s}}{\rm{e}}{\rm{c}}{\rm{h}}}^{2}y+1),\\ {V}_{2}(x,y) & = & \sum _{\sigma =x,y}[(8{m}_{0}-{\theta }_{0}^{2}-6){{\rm{s}}{\rm{e}}{\rm{c}}{\rm{h}}}^{2}\sigma -{m}_{0}({\theta }_{0}^{2}+10){{\rm{s}}{\rm{e}}{\rm{c}}{\rm{h}}}^{4}\sigma ]\\  &  & +\,{m}_{0}({\theta }_{0}^{2}+10){({\rm{s}}{\rm{e}}{\rm{c}}{\rm{h}}x{\rm{s}}{\rm{e}}{\rm{c}}{\rm{h}}y)}^{4},\\ {W}_{2}(x,y) & = & -{\theta }_{0}\sum _{\sigma =x,y}{\rm{s}}{\rm{e}}{\rm{c}}{\rm{h}}\sigma \tanh \sigma (7{m}_{0}{{\rm{s}}{\rm{e}}{\rm{c}}{\rm{h}}}^{2}\sigma +5),\\ {\varphi }_{2}(x,y) & = & \sqrt{{m}_{0}({\theta }_{0}^{2}+10)/g}{({\rm{s}}{\rm{e}}{\rm{c}}{\rm{h}}x{\rm{s}}{\rm{e}}{\rm{c}}{\rm{h}}y)}^{2}\exp [i{\theta }_{0}\sum _{\sigma =x,y}{\tan }^{-1}(\sinh \sigma )],\end{array}$$

where *μ* = 8.

*Case 3* (*α* = 3):

26a $$\begin{array}{ccc}{{\bf{m}}}_{3}(x,y) & = & {\rm{d}}{\rm{i}}{\rm{a}}{\rm{g}}({m}_{0}{{\rm{s}}{\rm{e}}{\rm{c}}{\rm{h}}}^{3}x+1,{m}_{0}{{\rm{s}}{\rm{e}}{\rm{c}}{\rm{h}}}^{3}y+1),\\ {V}_{3}(x,y) & = & -\frac{1}{4}\sum _{\sigma =x,y}[(4{\theta }_{0}^{2}+35){{\rm{s}}{\rm{e}}{\rm{c}}{\rm{h}}}^{2}\sigma -55{m}_{0}{{\rm{s}}{\rm{e}}{\rm{c}}{\rm{h}}}^{3}\sigma \\  &  & +\,{m}_{0}(4{\theta }_{0}^{2}+65){{\rm{s}}{\rm{e}}{\rm{c}}{\rm{h}}}^{5}\sigma ]+\frac{{m}_{0}}{4}(4{\theta }_{0}^{2}+65){({\rm{s}}{\rm{e}}{\rm{c}}{\rm{h}}x{\rm{s}}{\rm{e}}{\rm{c}}{\rm{h}}y)}^{5},\\ {W}_{3}(x,y) & = & -3{\theta }_{0}\sum _{\sigma =x,y}{\rm{s}}{\rm{e}}{\rm{c}}{\rm{h}}\sigma \tanh \sigma (3{m}_{0}{{\rm{s}}{\rm{e}}{\rm{c}}{\rm{h}}}^{3}\sigma +2),\\ {\varphi }_{3}(x,y) & = & \sqrt{{m}_{0}(4{\theta }_{0}^{2}+65)/(4g)}{({\rm{s}}{\rm{e}}{\rm{c}}{\rm{h}}x{\rm{s}}{\rm{e}}{\rm{c}}{\rm{h}}y)}^{5/2}\\  &  & \times \exp \{i{\theta }_{0}[{\tan }^{-1}(\sinh x)+{\tan }^{-1}(\sinh y)]\},\end{array}$$

where *μ* = 25/2.

## References

[1] Barton, G. *Introduction to Advanced Field Theory* (Wiley, New York, 1963).

[2] Bender CM, Boettcher S (1998). Real spectra in non-Hermitian Hamiltonians having PT symmetry. Phys. Rev. Lett..

[3] Dorey P, Dunning C, Tateo R (2001). Spectral equivalences, Bethe ansatz equations, and reality properties in PT-symmetric quantum mechanics. J. Phys. A: Math. Gen..

[4] Bender CM, Brody DC, Jones HF (2002). Complex extension of Quantum Mechanics. Phys. Rev. Lett..

[5] Bender CM (2007). Making sense of non-Hermitian Hamiltonians. Rep. Prog. Phys..

[6] Bender CM (2016). Rigorous backbone of PT-symmetric quantum mechanics. J. Phys. A: Math. Theor..

[7] Makris KG, El-Ganainy R, Christodoulides DN, Musslimani ZH (2011). PT symmetric periodic optical potentials. Int. J. Theor. Phys..

[8] Moiseyev, N. *Non*-*Hermitian Quantum Mechanics* (Cambridge Univ. Press, 2011).

[9] Kartashov YV, Malomed BA, Torner L (2014). Unbreakable PT symmetry of solitons supported by inhomogeneous defocusing nonlinearity. Opt. Lett..

[10] Ruschhaupt A, Delgado F, Muga JG (2005). Physical realization of PT -symmetric potential scattering in a planar slab waveguide. J. Phys. A: Math. Gen..

[11] El-Ganainy R, Makris KG, Christodoulides DN, Musslimani ZH (2007). Theory of coupled optical PT-symmetric structures. Opt. Lett..

[12] Berry MV (2008). Optical lattices with PT-symmetry are not transparent. J. Phys. A: Math. Theor..

[13] Klaiman S, Günther U, Moiseyev N (2008). Visualization of branch points in PT-Symmetric Waveguides. Phys. Rev. Lett..

[14] Longhi S (2009). Bloch oscillations in complex crystals with PT symmetry. Phys. Rev. Lett..

[15] Guo A (2009). Observation of PT-symmetry breaking in complex optical potentials. Phys. Rev. Lett..

[16] Rüter CE (2010). Observation of parity-time symmetry in optics. Nature Phys..

[17] Regensburger A (2012). Parity-time synthetic photonic lattices. Nature.

[18] Castaldi G, Savoia S, Galdi V, Alù A, Engheta N (2013). PT Metamaterials via Complex-Coordinate Transformation Optics. Phys. Rev. Lett..

[19] Hodaei H, Miri MA, Heinrich M, Christodoulides DN, Khajavikhan M (2014). Parity-time-symmetric microring lasers. Science.

[20] Peng B, Özdemir ŞK, Chen W, Nori F, Yang L (2014). Parity-time-symmetric whispering gallery microcavities. Nature Phys..

[21] Wimmer M (2015). Observation of optical solitons in PT-symmetric lattices. Nature Commun..

[22] Zhang Z (2016). Observation of parity-time symmetry in optically induced atomic lattices. Phys. Rev. Lett..

[23] Musslimani ZH, Makris KG, El-Ganainy R, Christodoulides DN (2008). Optical solitons in PT periodic potentials. Phys. Rev. Lett..

[24] Shi Z, Jiang X, Zhu X, Li H (2011). Bright spatial solitons in defocusing Kerr media with PT-symmetric potentials. Phys. Rev. A.

[25] Makris KG, El-Ganainy R, Christodoulides DN, Musslimani ZH (2008). Beam dynamics of PT-symmetric optical lattices. Phys. Rev. Lett..

[26] Driben R, Malomed BA (2011). Stability of solitons in parity-time-symmetric couplers. Opt. Lett..

[27] Abdullaev FKh, Kartashov YV, Konotop VV, Zezyulin DA (2011). Solitons in PT-symmetric nonlinear lattices. Phys. Rev. A.

[28] Li K, Kevrekidis PG (2011). PT-symmetric oligomers: Analytical solutions, linear stability, and nonlinear dynamics. Phys. Rev. E.

[29] Alexeeva NV, Barashenkov IV, Sukhorukov AA, Kivshar YS (2012). Optical solitons in PT-symmetric nonlinear couplers with gain and loss. Phys. Rev. A.

[30] Zezyulin DA, Konotop VV (2012). Nonlinear modes in finite-dimensional PT-symmetric systems. Phys. Rev. Lett..

[31] Nixon S, Ge L, Yang J (2012). Stability analysis for solitons in PT-symmetric optical lattices. Phys. Rev. A.

[32] Achilleos V, Kevrekidis PG, Frantzeskakis DJ, Carretero-González R (2012). Dark solitons and vortices in PT-symmetric nonlinear media: From spontaneous symmetry breaking to nonlinear PT phase transitions. Phys. Rev. A.

[33] Cartarius H, Wunner G (2012). Model of a PT-symmetric Bose-Einstein condensate in a PT-function double-well potential. Phys. Rev. A.

[34] Yan Z (2013). Complex-symmetric nonlinear Schrödinger equation and Burgers equation. Phil. Trans. R. Soc. A.

[35] Lumer Y, Plotnik Y, Rechtsman MC, Segev M (2013). Nonlinearly induced PT transition in photonic systems. Phys. Rev. Lett..

[36] Zhang X (2014). Discrete solitons and scattering of lattice waves in guiding arrays with a nonlinear PT-symmetric defect. Opt. Exp.

[37] D’Ambroise J, Kevrekidis PG, Malomed BA (2015). Staggered parity-time-symmetric ladders with cubic nonlinearity. Phys. Rev. E.

[38] Yan Z, Wen Z, Konotop VV (2015). Solitons in a nonlinear Schrödinger equation with PT-symmetric potentials and inhomogeneous nonlinearity: stability and excitation of nonlinear modes. Phys. Rev. A.

[39] Makris KG, Musslimani ZH, Christodoulides DN, Rotter S (2015). Constant-intensity waves and their modulation instability in non-Hermitian potentials. Nature Commun..

[40] Yan Z, Wen Z, Hang C (2015). Spatial solitons and stability in self-focusing and defocusing Kerr nonlinear media with generalized parity-time-symmetric Scarf-II potentials. Phys. Rev. E.

[41] Wen Z, Yan Z (2015). Dynamical behaviors of optical solitons in parity-time (PT) symmetric sextic anharmonic double-well potentials. Phys. Lett. A.

[42] Wen XY, Yan Z, Yang Y (2016). Dynamics of higher-order rational solitons for the nonlocal nonlinear Schrödinger equation with the self-induced parity-time-symmetric potential. Chaos.

[43] Li X, Yan Z (2017). Stability, integrability, and nonlinear dynamics of PT-symmetric optical couplers with cubic cross-interactions or cubic-quintic nonlinearities. Chaos.

[44] Kartashov YV, Konotop VV, Torner L (2015). Topological states in partially-PT-symmetric azimuthal potentials. Phys. Rev. Lett..

[45] Liu B, Li L, Mihalache D (2015). Vector soliton solutions in PT-symmetric coupled waveguides and their relevant properties. Rom. Rep. Phys..

[46] He Y, Zhu X, Mihalache D, Liu J, Chen Z (2012). Lattice solitons in PT-symmetric mixed linear-nonlinear optical lattices. Phys. Rev. A.

[47] Wang H (2015). Two-dimensional solitons in triangular photonic lattices with parity-time symmetry. Opt. Commun..

[48] He Y, Zhu X, Mihalache D (2016). Dynamics of spatial solitons in parity-time-symmetric optical lattices: a selection of recent theoretical results. Rom. J. Phys..

[49] Li P, Mihalache D, Li L (2016). Asymmetric solitons in parity-time-symmetric double-hump Scarf-II potentials. Rom. J. Phys..

[50] Kartashov YV, Hang C, Huang G, Torner L (2016). Three-dimensional topological solitons in PT-symmetric optical lattices. Optica.

[51] Hahn C (2016). Observation of exceptional points in reconfigurable non-Hermitian vector-field holographic lattices. Nature Commun..

[52] Burlak G, Garcia-Paredes S, Malomed BA (2016). PT-symmetric couplers with competing cubic-quintic nonlinearities. Chaos.

[53] Chen Y, Yan Z (2016). Solitonic dynamics and excitations of the nonlinear Schrödinger equation with third-order dispersion in non-Hermitian PT-symmetric potentials. Sci. Rep.

[54] Yan Z, Chen Y, Wen Z (2016). On stable solitons and interactions of the generalized Gross-Pitaevskii equation with PT- and non-PT-symmetric potentials. Chaos.

[55] Chen Y, Yan Z (2017). Stable parity-time-symmetric nonlinear modes and excitations in a derivative nonlinear Schrödinger equation. Phys. Rev. E.

[56] Suchkov SV (2016). Nonlinear switching and solitons in PT-symmetric photonic systems. Laser Photonics Rev..

[57] Konotop VV, Yang J, Zezyulin DA (2016). Nonlinear waves in PT-symmetric systems. Rev. Mod. Phys..

[58] Wannier GH (1937). The structure of electronic excitation levels in insulating crystals. Phys. Rev..

[59] Morrow RA, Brownstein KR (1984). Model effective-mass Hamiltonians for abrupt heterojunctions and the associated wave-function-matching conditions. Phys. Rev. B.

[60] van Roos O (1983). Position-dependent effective masses in semiconductor theory. Phys. Rev. B.

[61] Morrow RA (1987). Establishment of an effective-mass Hamiltonian for abrupt heterojunctions. Phys. Rev. B.

[62] Paul SF, Fouckhardt H (2001). An improved shooting approach for solving the time-independent Schrödinger equation for III/V QW structures. Phys. Lett. A.

[63] Konotop VV (1997). On wave propagation in periodic structures with smoothly varying parameters. J. Opt. Soc. Am. B.

[64] Midya B, Roy B, Roychoudhury R (2010). Position dependent mass Schrödinger equation and isospectral potentials: Intertwining operator approach. J. Math. Phys..

[65] Förster J, Saenz A, Wolff U (2012). Matrix algorithm for solving Schrödinger equations with position-dependent mass or complex optical potentials. Phys. Rev. E.

[66] Abdullaev FKh, Garnier J (1999). Solitons in media with random dispersive perturbations. Physica D.

[67] Burger S (2001). Superfluid and dissipative dynamics of a Bose-Einstein condensate in a periodic optical potential. Phys. Rev. Lett..

[68] Kramer M, Menotti C, Pitaevskii L, Stringari S (2003). Bose-Einstein condensates in 1D optical lattices - Compressibility, Bloch bands and elementary excitations. Eur. Phys. J. D.

[69] Eisenberg HS, Silberberg Y, Morandotti R, Aitchison JS (2000). Diffraction management. Phys. Rev. Lett..

[70] Longhi S (2009). Quantum-optical analogies using photonic structures. Laser Phot. Rev..

[71] Scarf FL (1958). New soluble energy band problem. Phys. Rev..

[72] Brazhnyi VA, Konotop VV (2004). Theory of nonlinear matter waves in optical lattices. Mod. Phys. Lett..

[73] Bagchi B, Quesne C (2000). sl(2, *C*) as a complex Lie algebra and the associated non-Hermitian Hamiltonians with real eigenvalues. Phys. Lett. A.

[74] Bagchi B, Quesne C, Znojil M (2001). Generalized Continuity equation and modified normalization in PT-symmetric quantum mechanics. Mod. Phys. Lett. A.

[75] Ahmed A (2000). Real and complex discrete eigenvalues in an exactly solvable one-dimensional complex PT-invariant potential. Phys. Lett. A.

[76] Trefethen, L. N. *Spectral Methods in Matlab* (SIAM, 2000).

[77] Shen, J. & Tang, T. *Spectral and High*-O*rder Methods with Applications* (Science Press, Beijing, 2006).

[78] Tsoy EN, Alaayarov IM, Abdullaev FKh (2014). Stable localized modes in asymmetric waveguides with gain and loss. Opt. Lett..

[79] Konotop VV, Zezyulin DA (2014). Families of stationary modes in complex potentials. Opt. Lett..

[80] Nixon S, Yang J (2016). Bifurcation of soliton families from linear modes in non-PT -symmetric complex potentials. Stud. Appl. Math..

[81] Wadati M (2008). Construction of parity-time symmetric potential through the soliton theory. J. Phys. Soc. Jpn..

[82] Kivshar, Y. S. & Agrawal, G. P. *Optical Solitons*: *From Fibers to Photonic Crystals* (Academic, San Diego, 2003).

[83] Malomed BA, Mihalache D, Wise F, Torner L (2005). Spatiotemporal optical solitons. J. Opt. B: Quantum Semiclassical Opt..

[84] Mihalache D (2015). Localized structures in nonlinear optical media: a selection of recent studies. Rom. Rep. Phys..

[85] Malomed BA, Torner L, Wise F, Mihalache D (2016). On multidimensional solitons and their legacy in contemporary atomic, molecular and optical physics. J. Phys. B: At. Mol. Opt. Phys..

[86] Ablowitz MJ, Musslimani ZH (2005). Spectral renormalization method for computing self-localized solutions to nonlinear systems. Opt. Lett..

[87] Kuznetsov EA, Rubenchik AM, Zakharov VE (1986). Soliton stability in plasmas and hydrodynamics. Phys. Rep..

[88] Yang, J. *Nonlinear Waves in Integrable and Nonintegrable Systems* (SIAM, Philadelphia, 2010).

